# A meta-analysis of impacts of immune response and infection on oxidative status in vertebrates

**DOI:** 10.1093/conphys/coac018

**Published:** 2022-04-06

**Authors:** David Costantini

**Affiliations:** Unité Physiologie Moléculaire et Adaptation, UMR 7221, Muséum National d’Histoire Naturelle, CNRS, CP32, 57 rue Cuvier 75005 Paris, France

**Keywords:** wildlife diseases, oxidative stress, life-history trade-offs, inflammation, ecoimmunology, antioxidant

## Abstract

Inferring from patterns observed in biomedical research, ecoimmunological theory predicts that oxidative stress is a ubiquitous physiological cost that contributes to generating variation in immune function between individuals or species. This prediction is, however, often challenged by empirical studies testing the relationship between immune response or infection and oxidative status markers. This points out the importance of combining ecological immunology and oxidative stress ecology to further our understanding of the proximate causes and fitness consequences of individual variation in health, and adaptability to natural and anthropogenic environmental changes. I reviewed evidence and performed phylogenetic meta-analyses of changes in oxidative status markers owing to either injection of an antigen or infection in captive and free-living vertebrates (141 studies, 1262 effect sizes, 97 species). The dataset was dominated by studies on fish, birds and mammals, which provided 95.8% of effect sizes. Both antigen injection and parasite exposure were associated with changes of oxidative status. There were significant effects of taxonomic class and experimental environment (captivity vs. wild). In contrast with my predictions, age category (young vs. adult), study design (correlational vs. experimental) and proxies of pace of life (clutch size, litter size, and body mass; for birds and mammals only) were negligible in this dataset. Several methodological aspects (type of immunostimulant, laboratory assay, tissue analysed) showed significant effects on both strength and direction of effect. My results suggest that alterations of oxidative status are a widespread consequence of immune function across vertebrates. However, this work also identified heterogeneity in strength and direction of effect sizes, which suggests that immune function does not necessarily result in oxidative stress. Finally, this work identifies methodological caveats that might be relevant for the interpretation and comparability of results and for the application in conservation programs.

## Introduction

Immunological function plays a crucial role in the maintenance of physiological homeostasis of living organisms, recognizing and protecting against infectious agents. Parasites and pathogens can have detrimental effects on survival and Darwinian fitness in animals. Infections are often associated with reductions in host energetic stores, harm to tissues, changes in behaviour, or impaired reproduction (e.g. Moore, 2002; Graham *et al*., 2011; Watson*,* 2013; Asghar *et al*., 2015; Sánchez *et al.,* 2018). Consequently, while individuals with well-functioning immune responses should be maintained by natural selection, poorly functioning ones should be counterselected. Thus, why is there large variation among individuals in immunological function? One answer to this question might lie with the factors that shape immune function, and the costs and benefits of an immune reaction, which in turn influence host–parasite relationships, disease outcomes and success of conservation actions (Mangalmurti and Hunter, 2020; Martin *et al*., 2011; Ohmer *et al*., 2021; Sheldon and Verhulst, 1996; Sorci *et al*., 2013; Sorci *et al*., 2017). For example, several authors proposed that physiological costs associated with an immune response might favour the maintenance of immunological variation because these costs are likely to vary with individual condition, environment, sex, age and high diversity of pathogens the hosts are exposed to (e.g. Klasing, 2004; Maizels and Nussey, 2013; Martin *et al*., 2008; Seppälä and Jokela, 2010; Sheldon and Verhulst, 1996).

Immune responses require various kinds of resources, which can lead to trade-offs with other physiological processes. Estimated energetic costs (percentage of increment in resting metabolic rate as compared to controls) of mounting an immune response during vaccination or sepsis may vary from 10% to 57% in humans or laboratory rodents (Lochmiller and Deerenberg, 2000). In birds, resting metabolic rates of several species increased from 5% to 15% following activation of humoral and cell-mediated immunities (Hasselquist and Nilsson, 2012). Apart from the dietary energy supply, nutritional modulation of the immune system is also reliant on the typology of micronutrients and macronutrients that are available. Growing evidence suggests that shortage of certain nutrients may impair immune functions, including reduced resistance to infections (e.g. Calder and Jackson, 2000; Gombart *et al*., 2020; Maggini *et al*., 2018; Wu *et al*., 2019).

Immune responses can also have other physiological effects. A hypothesis of immune-oxidative ecology states that perturbations of cellular oxidative status following an immune response might be costly for the organism (reviewed in Costantini, 2014). These perturbations, generally referred to as oxidative stress, may manifest as oxidative damage to biomolecules, oxidation of the key cellular antioxidant glutathione or changes in concentrations of antioxidant enzymes. It is increasingly recognized that oxidative stress might affect the risk of chronic diseases, cell senescence, fertility or longevity (reviewed in Costantini, 2014, 2019). Several lines of evidence provide strong support for a mechanistic link between immune response (including inflammation) and oxidative stress (Sorci and Faivre, 2009). For example, immune cells produce pro-oxidant chemicals (oxidative burst) to kill invading microorganisms (Sorci and Faivre, 2009). Pathogens with impaired antioxidant defences are more sensitive to phagocytic killing mediated by reactive oxygen species, supporting a role for pro-oxidant chemicals as microbicidal agents in living organisms (Halliwell and Gutteridge, 2015). Also, studies on knock-out mice (lacking a functional NADPH phagocyte oxidase or iNOS) suggested that individuals incapable of producing pro-oxidants are susceptible to severe bacterial or fungal infections (e.g. Mastroeni *et al*., 2000; Nathan and Shiloh, 2000). Beyond oxidative burst, immune response also raises oxygen consumption of cells (Hasselquist and Nilsson, 2012; Lochmiller and Deerenberg, 2000), which in turn might contribute to an increase in the generation of pro-oxidants.

Pro-oxidants produced by immune cells do not specifically target pathogens, so that they can also cause oxidative modifications to biomolecules (e.g. lipids, proteins, DNA), resulting in mutation, inflammation and tissue injury (Sorci and Faivre, 2009). Immune cells can themselves be damaged by their own pro-oxidants. For example, neutrophils with impaired glutathione reductase activity are more rapidly inactivated during phagocytosis than normal neutrophils (Halliwell and Gutteridge, 2015). Thus, hosts may experience trade-offs resulting from altered investment in immune function in relation to the need of regulating their oxidative status. However, empirical studies show that relationships between oxidative status markers, immunological reactions or infections can vary in strength and direction. For example, studies on bats and birds show large within- and among-species variation in the effects of immune response on oxidative status (e.g. Costantini and Møller, 2009; Cram *et al*., 2015; Fritze *et al*., 2019; Norte *et al*., 2018; Schneeberger *et al*., 2013; Sepp *et al*., 2012).

In this article, I reviewed evidence and used a phylogenetic meta-analytical approach to evaluate strength and direction of changes in oxidative status markers following immune response owing to antigen injection (any substance that is capable of stimulating an immune response; https://www.britannica.com/science/antigen) or exposure (natural or experimentally induced) to live parasites in vertebrates. I included free-living and captive populations for comparison to gain insight into what extent environmental variation in available resources can affect the physiological consequences of immune response. I expected that effect sizes would be larger for free-living animals than for captive animals because free-living animals might not readily recover from the costs of an immune response owing to stronger resource-based trade-offs. I also compared young (i.e. individuals sampled before sexual maturity) and adult individuals; I expected young individuals to suffer more oxidative stress than adults because they have immature antioxidant mechanisms (e.g. Fontagné *et al*., 2008; Surai, 2002). I also tested the effects of methodological aspects of study designs on effect sizes to assess the degree to which we need to refine our methods to improve interpretability and comparability of results. Finally, I tested the relationships between effect sizes and traits related to the pace of life in birds and in mammals, such as clutch size, litter size and body mass (e.g. Grainger and Levine, 2021; Sepp *et al*., 2018). I expected that species with faster paces of life would experience higher costs of immune response because of their higher metabolism and stronger investment in reproduction, which would come at a cost of antioxidant protection.

## Materials and methods

### Literature search

I performed a literature search (Supplementary Fig. S1) in the Web of Science All Databases for studies published between January 1950 and March 2021 about changes of oxidative status markers in relation to (i) injection of antigens, (ii) experimentally induced infections with live parasites (e.g. injection of a pathogen into the peritoneum) and (iii) comparisons between naturally infected and non-infected individuals in both captive and wild vertebrates, with the exclusion of laboratory mice or highly domesticated taxa because artificial selection might generate phenotypic responses that are not comparable to those observed in wild animals. Relying on the keywords used for the search on online databases, additional searches were carried out through the screening of cited literature in the selected articles from which data were taken. Finally, I have also consulted Google Scholar and PubMed, but I could not find any additional articles. As keywords, I used the following terms: ((immu^*^ OR pathog^*^ OR infect^*^ OR parasit^*^) AND (antiox^*^ OR oxida^*^) NOT (clinic^*^ OR hospital OR mouse OR chicken OR human OR industry OR plant^*^ OR agric^*^ OR rat OR patient OR crab OR shrimp OR in vivo OR cell culture OR in vitro OR mutant)). The search was refined by selecting the following categories in WoS, which enabled me to collect only papers that fell into one of these categories: ecology or veterinary sciences or fisheries or marine freshwater biology or evolutionary biology or parasitology or behavioural sciences or environmental sciences or zoology or ornithology or biodiversity conservation. After the completion of this search, I screened all papers applying these following exclusion criteria: (i) studies that measured only the expression of antioxidant genes because I was interested in the biochemical outcomes, such as oxidation products or concentration of given antioxidants, which provide a more direct representation of cellular oxidative status; (ii) studies that only quantified the free radical generation because assays are not specific for a given free radical nor do they allow to infer about oxidative damage (e.g. Jaguezeski *et al*., 2020; Tobler *et al*., 2011); and (iii) studies that were not carried out on vertebrates.

I categorized oxidative status markers as follows: (i) oxidation [e.g. protein carbonyls, dROMs (reactive oxygen metabolites), MDA (malondialdehyde), TBARS (thiobarbituric acid reactive substances), GSSG (oxidized glutathione)]; (ii) non-enzymatic antioxidants [e.g. GSH (reduced glutathione), total thiols, assays of antioxidant capacity (OXY)]; and (iii) antioxidant enzymes [e.g. CAT (catalase), GST (glutathione-S-transferase), GPx (glutathione peroxidase), SOD (superoxide dismutase), GR (glutathione reductase)]. I also categorized the oxidative status markers by the typology of laboratory assay used (e.g. TBARS, MDA by HPLC, d-ROMs, KRL, GSH) and by tissue (e.g. blood, brain, liver, muscle; Supplementary Table S1). From each paper, I also extracted information about age category of experimental animals, which included young (before sexual maturity) and adult individuals, species, experimental environment (captivity vs. wild), type of parasite (e.g. worm, virus; Supplementary Table S2), type of antigen injection (e.g. LPS, PHA) and time elapsed from antigen injection or parasite exposure to final tissue sampling. Information on sex was available for a small number of studies, so that it could not be considered in subsequent analyses. I used all these metrics as moderators in the models.

### Calculation of effect sizes

I used the *compute.es* package (del Re, 2013) in R version 4.0.5 to calculate the standardized effect size Hedges’ g and its sampling variance from descriptive statistics (means and standard deviations) and sample sizes or test statistics (e.g. *t*-values or F ratios). If data were not reported in the text or tables, I extracted them from graphs using the software GetData Graph Digitizer (Fedorov, 2014). When extraction of data was not possible, I contacted first and/or last authors. I made requests for 44 articles; only 7 did not respond, and their articles were excluded from the work. In total, I extracted 1262 effect sizes, of which 1209 were derived from descriptive statistics.

In the statistical analyses, as dependent variables, I used the unsigned and the signed effect sizes in separate models, respectively. Unsigned effect sizes indicate the strength of the difference in a given marker between groups (i.e. the magnitude of the effect). However, unsigned effect sizes do not provide information on the direction of the change, e.g. if oxidative damage increased or decreased in immune-challenged individuals. This is relevant information to make inferences about oxidative stress. Thus, I also used signed effect sizes. To do so, effect sizes were given a (i) positive value when either oxidative damage was higher or a given antioxidant was lower in immunostimulated or infected animals than in control animals and (ii) a negative value when either oxidative damage was lower or a given antioxidant was higher in immunostimulated or infected animals than in control animals. A positive effect size would imply higher biochemical oxidative stress (for a similar approach, see Costantini and Møller, 2009).

### Phylogenetic meta-analyses

Analyses were carried out following Nakagawa and Cuthill (2007), Hadfield (2010), Nakagawa and Santos (2012), Garamszegi (2014) and Nakagawa *et al*. (2022). Note that, as the distribution of unsigned effect sizes is a folded normal distribution (Morryssey, 2016a, 2016b), I did not derive effect sizes because, while contrasts between estimates are reliable because they are placed on the same scale, magnitudes can be inflated. Moreover, for the goals of this work, signed effect sizes were granted as more relevant for ecological and conservation sciences.

First, I ran a phylogenetic meta-analysis in R using the package *MCMCglmm* to calculate the meta-analytical mean for signed effect sizes for the whole dataset. I also included the sampling variance associated with each effect size inserting the *mev* argument of the *MCMCglmm* package. To account for pseudo-replication, I included multiple random factors: species (non-independence of multiple effect sizes obtained from the same species), phylogenetic relatedness, article (non-independence of effect sizes from the same study), tissue where a given marker was analysed (variation in tissues analysed across studies), laboratory assay (variation in assays performed across studies) and type of immunostimulant (injection of an antigen or exposure to a given parasite). The phylogeny used to compute a phylogenetic covariance matrix was obtained from http://www.timetree.org. Eight species were replaced with closely related species in order to build the tree; the exclusion of these species did not affect outcomes (Supplementary Table S4), so that they were retained in the final models. The phylogenetic signal was calculated as lambda (λ) using the package *phytools*.

Then, I conducted two phylogenetic meta-analyses on the full dataset including the unsigned and the signed effect sizes as response variables, separately. In both models, I included the taxonomic class, the experimental environment (captivity or wild), the age category (young, adult or mix for studies that used a pool of young and adults), the marker category (oxidation, antioxidant enzyme or non-enzymatic antioxidant) and the study design (experimental or non-experimental) as fixed explanatory variables (termed moderators in meta-analysis). Note that studies carried out on captive and wild animals included both non-experimental (correlative) and experimental studies. I also included the sampling variance associated with each effect size using the *mev* argument. As random factors, I included the same factors as those used to calculate the meta-analytical means.

In a second set of analyses, I focussed on the effects of methodological approaches, which vary largely among studies. I ran meta-analyses on sub-sets of data including the following factors: hours elapsed from immunostimulation to final sampling (included as a covariate) and the random factors (species + phylogeny + article + tissue + assay + type of immunostimulant); hours elapsed from the start of infection to final sampling (included as a covariate) and the random factors (species + phylogeny + article + tissue + assay + type of immunostimulant); type of immunostimulant as fixed factor for the categories with sample sizes >40 and the random factors (species + phylogeny + article + tissue + assay); laboratory assay of oxidative status marker as fixed factor for the categories with sample sizes >40 and the random factors (species + phylogeny + article + tissue + type of immunostimulant); and tissue analysed as fixed factor for the categories with sample sizes >40 and the random factors (species + phylogeny + article + assay + type of immunostimulant). In each of these additional meta-analyses, I always included the sampling variance associated with each effect size using the *mev* argument.

Finally, I ran two separate meta-analyses for birds and mammals, respectively, to test the effects of proxies of pace of life (clutch size for birds and litter size for mammals; body mass for both birds and mammals; data obtained from https://worldspecies.org/). In each of these two models, I included the experimental environment, the age, the marker and the study design as additional fixed factors, and the sampling variance associated with each effect size using the *mev* argument. As random factors, I included species, article, laboratory assay, tissue, type of immunostimulant and phylogeny.

I ran all the Markov chain Monte Carlo models for 150 000 iterations, with sampling every 50 iterations with the first 50 000 removed as burn-in (e.g. Sanders, Frago *et al*., 2021). Then I tested the autocorrelation using the function *autocorr.diag*: lag values were always <0.1, indicating that autocorrelation between subsequent iterations was negligible. Convergence of the chains was also inspected visually to ensure that there were no trends in the chain and that posterior distributions were not skewed (for full datasets, see Supplementary Figs S2 and S3). Since I did not have any a priori knowledge on the distribution of data, I used flat priors: V = 1 and nu = 0.002. To assess the influence of the informativeness of the priors (nu) on the models, I repeated the analyses with different priors, with no detectable effect on the results. I reported significance as the pMCMC. Effect sizes were considered to be small when g = 0.2 (1% of the variance explained by a factor with a g value of 0.2), intermediate when g = 0.5 (9% of the variance explained by a factor with a g value of 0.5) or large when g = 0.8 (25% of the variance explained by a factor with a g value of 0.8) as described by Cohen (1988) and Møller and Jennions (2002).

Publication bias was assessed by examining funnel plots of effect sizes against the log_10_ of sample sizes for the full dataset (Møller and Jennions, 2001) and by performing full multi-level meta-regressions including log_10_ of sample sizes and the effective sample size as described by Nakagawa *et al*. (2022), respectively. In so doing, the publication bias test is conditioned on all the various sources affecting heterogeneity (i.e. fixed effects and random effects included in the model for the full dataset). Additionally, I tested for the presence of a time trend in signed effect sizes by fitting a continuous year variable in an MCMC model that included the taxonomic class, the experimental environment, the age category, the marker and the study design as fixed explanatory variables; species + phylogeny + article + tissue + assay + type of immunostimulant as random effects; and the sampling variance associated with each effect size using the *mev* argument.

## Results

### Dataset description

My final dataset included 141 studies that involved 1262 effect sizes from 97 species (Table 1): 4 amphibians (44 effect sizes), 34 birds (253 effect sizes), 33 fish (790 effect sizes), 22 mammals (166 effect sizes) and 4 reptiles (9 effect sizes). There were 803 and 459 effect sizes from captivity and wild studies, respectively; 766 and 496 from experimental and non-experimental studies, respectively; 631, 247 and 384 from enzymatic antioxidants, non-enzymatic antioxidants and oxidation markers, respectively; 490, 503 and 269 from studies using adults, young and both adults and young (mix), respectively. The time elapsed from the injection of an antigen to final sampling varied from 6 to 1440 hours and was available for 49 studies; the time elapsed from the start of infection to final sampling varied from 6 to 7392 hours and was available for 78 studies.

**Table 1:** List of 97 species and 141 articles included in the meta-analysis

Taxonomic class	Species	Article
Amphibians	*Amietophrynus regularis*	Akinsanya *et al*., 2020
Amphibians	*Elachistocleis bicolor*	Ghirardi *et al*., 2020
Amphibians	*Lithobates pipiens*	Marcogliese *et al*., 2021
Amphibians	*Physalaemus albonotatus*	Ghirardi *et al*., 2018
Birds	*Accipiter gentilis*	Hanssen *et al*., 2013
Birds	*Acrocephalus sechellensis*	van de Crommenacker *et al*., 2012
Birds	*Agelaius phoeniceus*	Schoenle *et al*., 2017
Birds	*Alectoris rufa*	Perez-Rodriguez *et al*., 2008; Mougeot *et al*., 2009
Birds	*Arachnothera longirostra*	Messina *et al*., 2022
Birds	*Carduelis chloris*	Hõrak *et al*. 2006, 2007; Sepp *et al*., 2012; Pap *et al*., 2014
Birds	*Colinus virginianus*	Baylor and Butler, 2019; Armour *et al*., 2020
Birds	*Columba livia*	Razavi *et al*., 2016
Birds	*Diomedea exulans*	Costantini *et al*. 2015b
Birds	*Falco naumanni*	Rodríguez *et al*. 2014
Birds	*Falco tinnunculus*	Costantini and Dell’Omo 2006; Casagrande *et al*., unpublished results
Birds	*Ficedula hypoleuca*	López-Arrabé *et al*. 2015
Birds	*Fregata magnificens*	Sebastiano *et al*., 2017, 2018
Birds	*Geopelia cuneata*	Casagrande *et al*., 2012
Birds	*Haemorhous mexicanus*	Giraudeau *et al*., 2014; Zhang *et al*., 2021
Birds	*Haliaeetus albicilla*	Hanssen *et al*., 2013
Birds	*Lagopus lagopus*	Mougeot *et al*., 2010
Birds	*Malacocincla malaccensis*	Messina *et al*., 2022
Birds	*Malacocincla sepiaria*	Messina *et al*., 2022
Birds	*Malacopteron cinereum*	Messina *et al*., 2022
Birds	*Parus major*	Losdat *et al*., 2011; De Coster *et al*., 2012; Isaksson *et al*., 2013; Marri and Richner, 2015; Wegmann *et al*., 2015; Delhaye *et al*., 2016; De Angeli *et al*., 2017; Maronde *et al*., 2018
Birds	*Passer domesticus*	Pap *et al*., 2011; Broggi *et al*., 2016
Birds	*Perdix perdix*	Vitula *et al*., 2011
Birds	*Phasianus colchicus*	Orledge *et al*., 2012
Birds	*Plocepasser mahali*	Cram *et al*., 2015
Birds	*Serinus canaria*	Delhaye *et al*., 2018
Birds	*Stachyris poliocephala*	Messina *et al*., 2022
Birds	*Sturnus vulgaris*	Serra *et al*., 2012; Costantini *et al*. 2015a
Birds	*Sula nebouxii*	Torres and Velando, 2007
Birds	*Taeniopygia guttata*	Alonso-Alvarez *et al*., 2004; Bertrand *et al*., 2006; Casasole *et al*., 2016
Birds	*Trichastoma bicolor*	Messina *et al*., 2022
Birds	*Tricholestes criniger*	Messina *et al*., 2022
Birds	*Turdus merula*	Hau *et al*., 2015; Norte *et al*., 2018
Birds	*Zenaida macroura*	Sweazea *et al*., 2015
Fish	*Anguilla anguilla*	Schneebauer *et al*., 2016
Fish	*Argyrosomus regius*	Peixoto *et al*., 2017
Fish	*Astyanax aeneus*	Espinal-Carrion and López-Lopez 2010
Fish	*Carassius gibelio*	Xu *et al*., 2020
Fish	*Channa argus*	Li *et al*., 2020; Zhu *et al*., 2020
Fish	*Clarias gariepinus*	Adeyemi, 2014
Fish	*Ctenopharyngodon idella*	Kim, Ke, and Zhang, 2009; Baldissera *et al*., 2019; Zhao *et al*., 2019
Fish	*Cyprinus carpio*	Dautremepuits *et al*., 2003; Chen *et al*., 2020; Giri *et al*., 2020; Sun *et al*., 2020
Fish	*Gasterosteus aculeatus*	Le Guernic *et al*., 2016
Fish	*Gobio occitaniae*	Petitjean *et al*., 2021a,b
Fish	*Labeo rohita*	Roy *et al*., 2019; Harikrishnan *et al*., 2020; Pal *et al*. 2020
Fish	*Lutjanus peru*	Guzmán-Villanueva *et al*., 2014
Fish	*Megalobrama amblycephala*	Abasubong *et al*., 2018; Geng *et al*., 2019
Fish	*Merlangius merlangus*	Skuratovskaya *et al*., 2015
Fish	*Misgurnus anguillicaudatus*	Yun *et al*., 2019
Fish	*Oncorhynchus mykiss*	Tkachenko *et al*., 2016; Azimzadeh and Amniattalab, 2017; Şahan *et al*. 2017
Fish	*Oreochromis niloticus*	Ali *et al*., 2011; Zahran and Risha, 2013; Zahran *et al*., 2018; Zahran *et al*., 2019; Abdelkhalek *et al*., 2020; El-Son *et al*., 2020; Ren *et al*., 2020
Fish	*Pangasianodon hypophthalmus*	Kumar *et al*., 2017
Fish	*Paralichthys olivaceus*	Kim *et al*., 2020
Fish	*Pelteobagrus fulvidraco*	Liu *et al*., 2020
Fish	*Perca flavescens*	Marcogliese *et al*., 2005; Marcogliese *et al*., 2010
Fish	*Pimephales promelas*	Stumbo *et al*., 2012
Fish	*Ponticola eurycephalus*	Skuratovskaya *et al*., 2020
Fish	*Rhamdia quelen*	Bello *et al*. 2000; Garcia *et al*., 2011; Tancredo *et al*., 2015; Baldissera *et al*., 2017; da Rosa *et al*., 2019
Fish	*Rutilus rutilus*	Jolly *et al*., 2014
Fish	*Salmo salar*	Du *et al*., 2015
Fish	*Salmo trutta*	Kurhalyuk *et al*., 2009; Tkachenko *et al*., 2014; Kurhalyuk and Tkachenko, 2011; Kurhalyuk *et al*., 2011; Hoogenboom *et al*., 2012; Stauffer *et al*., 2017; Tkachenko *et al*., 2011
Fish	*Scophthalmus maximus*	Rodríguez-Quiroga *et al*., 2017
Fish	*Seriola quinqueradiata*	Ito *et al*., 2000
Fish	*Siganus oramin*	Jiang *et al*., 2017
Fish	*Sparus aurata*	Balasch *et al*., 2019.
Fish	*Squalius cephalus*	Molbert *et al*., 2020
Fish	*Zosterisessor ophiocephalus*	Skuratovskaya *et al*., 2020
Mammals	*Bubalus bubalis*	Allaam *et al*., 2014
Mammals	*Camelus dromedarius*	Saleh *et al*., 2009; Heidarpour *et al*., 2012; Azma *et al*., 2015; Amin *et al*., 2020
Mammals	*Carollia perspicillata*	Schneeberger *et al*., 2013
Mammals	*Equus hemionus*	Costantini *et al*., 2018
Mammals	*Equus quagga*	Costantini *et al*., 2018
Mammals	*Macaca mulatta*	Srivastava *et al*., 1992
Mammals	*Mandrillus sphinx*	Charpentier *et al*., 2019
Mammals	*Meriones unguiculatus*	Dkhil *et al*., 2013; Tonin *et al*., 2014
Mammals	*Mesocricetus auratus*	Charoensuk *et al*., 2011; Sobroza *et al*., 2014
Mammals	*Microtus arvalis*	Devevey *et al*., 2010.
Mammals	*Myotis brandtii*	Lilley *et al*., 2014
Mammals	*Myotis daubentonii*	Lilley *et al*., 2014
Mammals	*Myotis lucifugus*	Moore *et al*., 2013
Mammals	*Myotis myotis*	Fritze *et al*., 2019, 2021
Mammals	*Myotis mystacinus*	Lilley *et al*., 2014
Mammals	*Myotis vivesi*	Hernández-Arciga *et al*., 2020
Mammals	*Octodon degus*	Ramirez-Otarola *et al*., 2019, 2021
Mammals	*Oryctolagus cuniculus*	Pacios-Palma *et al*., 2018
Mammals	*Pipistrellus nathusii*	Voigt *et al*., 2020.
Mammals	*Plecotus auritus*	Lilley *et al*., 2014
Mammals	*Sus scrofa*	Gassó *et al*., 2016
Mammals	*Urocitellus columbianus*	Roth *et al*., 2019
Reptiles	*Chelonia mydas*	da Silva *et al*., 2016
Reptiles	*Ctenophorus pictus*	Tobler *et al*., 2015
Reptiles	*Uta stansburiana*	Alaasam *et al*., 2021
Reptiles	*Thamnophis sirtalis*	Neuman-Lee *et al*., 2019

### Results of full dataset

**Figure 1:**
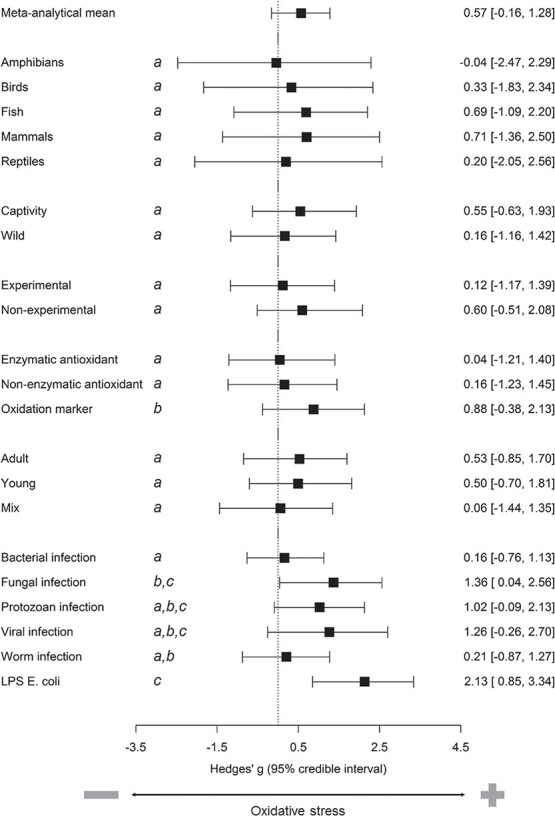
Summary of the evidence for changes in oxidative status markers in individuals challenged with an immunostimulant (antigen or live parasite). Forest plots show predictions of models for the overall meta-analytical mean with 95% credible intervals and for the effects of moderators on this mean. Outcomes of contrasts between levels are shown as letters: shared letters (e.g. both levels with a) indicate that the two levels of a given moderator do not differ significantly. The dataset included 141 studies that involved 1262 effect sizes from 97 species.

Meta-analytical mean of signed effect sizes was intermediate and the 95% CI included zero (0.567, −0.160/1.281, *P*_MCMC_ = 0.099; Fig. 1). Unsigned effect sizes were significantly larger in studies on captive animals than in those on free-living animals and marginally smaller in birds than in fish or mammals (Supplementary Table S3). By contrast, age category, marker category or study design did not explain variation in effect sizes (Supplementary Table S3). The lambda phylogenetic signal was 0.0441 (posterior mode = 0.0015, HPD interval = 0.0001/0.2132). Signed effect sizes were significantly larger for oxidation markers than for enzymatic or non-enzymatic antioxidant markers (Fig. 1; Supplementary Table S3). Taxonomic class, age category, experimental environment and study design did not explain variation in effect sizes. The lambda phylogenetic signal was 0.1377 (posterior mode = 0.0019, HPD interval = 0.00005/0.4018). Results of models for both unsigned and signed effect sizes were unchanged if very large effect sizes (>10, *n* = 48) were removed from the models (Supplementary Table S5).

### Effect of sampling time

Models included 158 and 518 effect sizes for antigen type and for infection with a parasite, respectively. Results show that both unsigned and signed effect sizes were not related to the time elapsed from antigen injection to final sampling nor to the time elapsed since the start of the infection until the final sampling, with or without centering of the covariate (Supplementary Table S6).

### Effects of immunostimulant type, laboratory assay and tissue

The most common types of immunostimulant in the dataset (with a number of effect sizes >40) were bacterial infection (428), fungal infection (102), LPS *Escherichia coli* injection (94), protozoan infection (180), viral infection (59) and worm infection (170), which represented altogether 81.9% of effect sizes. The model testing the effects of immunostimulant type (1033 effect sizes) shows that unsigned effect sizes were similar among these six types of immunostimulant, with the exception of LPS *E. coli* injection whose effect size was significantly larger than that of worm infection (Supplementary Table S7). Signed effect sizes of LPS *E. coli* injection and of fungal infection were the only types of immunostimulant to differ significantly from zero (Fig. 1; Supplementary Table S6). Effect sizes of LPS *E. coli* injection were significantly larger than bacterial infection and worm infection; effect sizes of fungal infection were significantly larger than bacterial infection (Supplementary Table S7).

**Figure 2:**
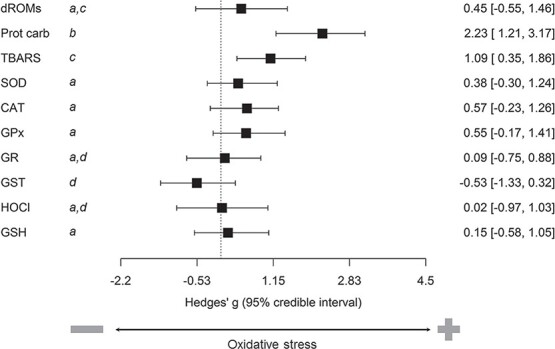
Summary of the evidence for effect of laboratory assay of oxidative status markers. Forest plot show predictions of models (mean with 95% credible intervals). Outcomes of contrasts between levels are shown as letters: shared letters (e.g. both levels with a) indicate that the two levels of a given moderator do not differ significantly.

The model testing the laboratory assay effect (1093 effect sizes) included 10 assays with a number of effect sizes >40, which represented altogether 86.6% of effect sizes: three assays of oxidative damage [dROMs (52), TBARS (218) and protein carbonyls (41)], two non-enzymatic antioxidants [OXY (49), GSH (102)] and five enzymatic antioxidants [SOD (189), GPx (99), catalase (177), GST (87) and GR (79)]. Effect size of (i) protein carbonyls was significantly larger than that of all other assays with the exceptions of GPx and GST, (ii) GST was significantly larger than that of SOD and of OXY and (iii) GPx was significantly larger than that of OXY, SOD and GR (Supplementary Table S8). Signed effect sizes were significantly different from zero for protein carbonyls and TBARS (Fig. 2; Supplementary Table S8). Effect size of (i) protein carbonyls was significantly larger than that of all other assays; (ii) TBARS was significantly larger than that of CAT, GPX, GSH, GST, OXY, SOD and GR; and (iii) GST was significantly smaller than that of CAT, dROMs, GPx, GSH and SOD (Supplementary Table S8).

**Figure 3:**
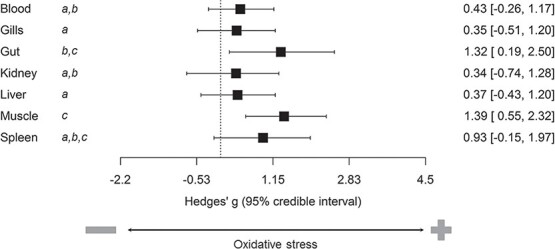
Summary of the evidence for effect of tissue analysed. Forest plot show predictions of models (mean with 95% credible intervals). Outcomes of contrasts between levels are shown as letters: shared letters (e.g. both levels with a) indicate that the two levels of a given moderator do not differ significantly.

Finally, the model testing the effect of tissue (1183 effect sizes) included blood (499), gills (120), gut (52), kidney (42), liver (294), muscle (132) and spleen (44), which were the most common tissues analysed, representing altogether 93.7% of effect sizes. Unsigned effect size of (i) gut was significantly larger than that of any other tissue, (ii) blood was significantly smaller than liver and muscle and (iii) kidney was significantly smaller than that of liver and muscle (Supplementary Table S9). Signed effect sizes were significantly different from zero for gut and muscle (Fig. 3; Supplementary Table S9). Effect size of (i) gut was significantly larger than that of gills and liver and (ii) muscle was significantly larger than that of blood, gills, liver and kidney (Fig. 3; Supplementary Table S9).

### Pace of life

Models testing the effects of pace of life included 253 and 166 effect sizes for birds and mammals, respectively. Results showed that neither clutch size nor body mass were significantly related to both unsigned and signed effects sizes in birds (Supplementary Table S10). Similarly, in mammals, results showed that neither litter size nor body mass were significantly related to both unsigned and signed effect sizes (Supplementary Table S10).

### Publication bias

The plot calculated on the full dataset appears in the shape of a ‘funnel’ (Supplementary Fig. S4). A meta-regression model showed that signed effect sizes were not related to the log_10_ of sample size (estimate/ci.lb/ci.ub: −0.169/−0.864/0.499, pMCMC = 0.623) nor to the effect sample size (estimate/ci.lb/ci.ub: 0.00002/−0.00483/0.00491). There was no time trend in effect sizes for both unsigned (publication year: postmean and 95% CI: −0.006, −0.047/0.036) and signed (publication year: postmean and 95% CI: −0.021, −0.080/0.031) effect sizes. These results are indicative of absence of publication bias.

## Discussion

Inferring from the patterns observed in humans and laboratory rodents, ecoimmunology predicts that alterations of the cellular oxidative status are one relevant physiological cost of immune function that might impinge on fitness. The outcomes of these phylogenetic meta-analyses suggest that immune response owing to both antigen injection and exposure to a parasite cause change of oxidative status in vertebrates. These meta-analyses also show that there is substantial variation in the direction and strength of effects across studies. Thus, a change in oxidative status did not necessarily result in strong oxidative stress. These results were robust for variation among studies in laboratory assays used to assess the oxidative status, the tissue analysed or the type of immunostimulant, which were all controlled for in the models.

Immune response caused significant and strong changes of oxidative status markers across all five classes of vertebrates. The directionality of these changes was similar across vertebrates, but it was very variable within each class. These results suggest that immune response did not always necessarily result in increased oxidative damage or decreased antioxidant levels in any taxonomic class. My sample size was not, however, sufficiently high to draw robust conclusions about amphibians and reptiles. The dataset was actually dominated by studies on fish, birds and mammals, which provided 95.8% of effect sizes. This similarity in effect sizes among these three taxonomic classes suggests that endothermy or reproductive mode might not have a strong influence on general patterns of oxidative status variation in immune-challenged vertebrates. A recent meta-analysis found that relationships between infection and metrics of body condition (indicative of energetic status) were generally more strongly negative among endotherms than ectotherms, possibly because warm-blooded animals pay a metabolic cost of body temperature regulation and offer a constant endogenous environment that might favour parasite replication (Sánchez *et al.,* 2018). By contrast, oxidative status might not be strongly linked to these metabolic costs because (i) immune function also affects oxidative status through pathways that are independent from energetic balance (e.g. oxidative burst in leukocytes; Sorci and Faivre, 2009) and (ii) increased metabolic demands caused by immune response do not induce proportional increases in generation of pro-oxidants (e.g. through the uncoupling between mitochondrial energy production and free radical generation; Herrero and Barja, 1997). Costs of immunity, however, can be highly variable depending on the type of infection and the tolerance/resistance of the infected species and show up in different ways (e.g. the emergence of immunopathologies). The results of my meta-analyses actually show heterogeneity in effect sizes, which might be due to some degree to this variation in costs among types of immunostimulant, masking any relationships between proxies of pace of life and effect sizes.

Contrary to my expectations, unsigned effect sizes were significantly larger in studies on captive animals than in those on free-living animals. This result does not confirm the preliminary observations from Costantini and Møller (2009) indicating that the oxidative status homeostasis of free-living birds might be less resistant or resilient to an immune challenge than that of captive birds. This greater sensitivity of captive animals might be attributable to a high investment of resources into the immune response. As compared to wild animals, laboratory animals are not subject to a full complement of natural and sexual selection pressures. For example, food is provided *ad libitum* and metabolic costs for foraging or mating are kept at minimum. Trade-offs would therefore be less stringent under the conditions of relaxed selection in captivity. As a consequence, captive animals might have invested more resources in the immune response, which came at a cost for the oxidative status homeostasis. On the other hand, captive animals might also experience different parasite loads or suboptimal conditions, such as space constraints that favour social stress, inadequate nutrient intakes and stressful abiotic conditions (e.g. temperature, humidity or light regimes), that might further impair physiological homeostasis. Also, captive and wild co-specific individuals might rely on different immune strategies that could generate heterogeneity in effect sizes. For example, Seeber *et al*. (2020) found that energetically cheap markers of constitutive innate immunity were lower in captive than in wild zebras, whereas energetically costly markers of the induced innate immunity were more highly expressed in captive zebras. Flies *et al*. (2015) show that wild hyenas have significantly higher serum antibody concentrations, natural antibodies and autoantibodies than do captive hyenas, but similar bacterial killing capacity of sera between captive and wild hyenas. Thus, captive animals might not be always adequate models to estimate the costs of immune function in wild animals.

This dataset included both correlational and experimental studies. A larger effect in correlational studies would indicate that co-variation between effect sizes and a given moderator might not be causal (e.g. Simons *et al*., 2012). This aspect of research design was negligible in this dataset. Effect sizes were indeed similar between experimental and correlational studies, indicating that differences in oxidative status between naturally infected and non-infected individuals in observational studies might reflect a cause-effect relationship. This result is not in line with a meta-analysis that found that relationships between infection and metrics of body condition were more strongly negative for experimental (e.g. parasite removal or manipulation of host nutritional state) than for observational studies (Sánchez *et al.,* 2018). As discussed previously, alterations of oxidative status can also be induced by factors (e.g. oxidative burst) that operate independently from energetic status. Thus, relying on a single category of metrics to estimate costs of immune function might not be adequate for capturing the complexity of the multifaceted nature of these physiological effects.

The present meta-analyses also show that young and adult individuals have similar effect sizes. In vertebrates, individuals are born with the innate arm of the immune system, which matures and acquires memory with time, and then declines in old age (reviewed in Georgountzou and Papadopoulos, 2017). Similarly, levels of endogenous antioxidants are low in young and increase from birth to adulthood (e.g. Surai, 2002; Fontagné *et al*., 2008; reviewed in Costantini, 2014). This result suggests that maturations of immune function and of oxidative status might be synergistically co-programmed and canalized to some degree in order to balance costs and benefits. This comparison between young and adult individuals is, however, limited by the lack of control for individual age (e.g. middle-age adults vs. senescent adults) that might affect senescence of the immune function (Peters *et al*., 2019) or control of metabolic demands of growth in young individuals, which have a significant effect on oxidative stress (Smith *et al*., 2016).

This work also identifies methodological caveats that might be relevant for the interpretation and comparability of results. Both the time elapsed from (i) the injection of an antigen to final sampling and (ii) the start of infection with a living parasite to final sampling did not have a significant effect on effect sizes in the present dataset. By contrast, these meta-analyses detected significant effects of the type of immunostimulant, the laboratory assay of oxidative status and the tissue analysed. LPS *E. coli* injection and fungal infection generated strong effects as compared to other immunostimulants. It is becoming clear that parasites differ in their modes of interaction with the host immune defences, including oxidative burst and antioxidant levels (Paiva and Bozza, 2014). For example, different types of viruses sustain their replication cycle by promoting a pro-oxidant environment in the infected cell, actively interfering with its redox homeostasis and antioxidant defence systems (Camini *et al*., 2017). By contrast, parasites of the genus *Leishmania* may inhibit the production of pro-oxidants by phagocytes (Panaro *et al*., 1998), while helminths can release a number of metabolites with pro-oxidant properties that may result in mutagenic and carcinogenic effects (Brindley *et al*., 2015). Sampling design might also generate variation in effect sizes among types of immunostimulants. A total of 11 out of 16 LPS studies of which information on sampling was available collected samples for oxidative status analyses within 24 hours from antigen injection. This is because LPS studies aim to test acute inflammatory effects of innate immune response to a simulated bacterial infection. By contrast, sampling in studies that infected the animals experimentally with live parasites was carried out on average many days after parasite injection (e.g. 392 hours for bacterial infection; 1269 hours for worm infection). Thus, while LPS studies provide relevant information about acute effects, they might not be appropriate to infer adequately about long-term effects on oxidative status associated with prolonged infections. Hosts might also detoxify from parasites or start to tolerate them better and have a milder response because the parasite replication is dampened. Dose of antigen injected into the animal and parasite load might be two additional relevant factors that generate variation in effect sizes. I could not test for their effects because of the limited information available. Comparisons of groups of animals that were experimentally injected with different amounts of a given pathogen showed that oxidative damage is not always higher in those animals that were exposed to higher parasite concentrations, possibly because the amount injected weakly reflected the actual individual parasite load (e.g. Adeyemi, 2014; Armour *et al*., 2020; Sun *et al*., 2020). On the other hand, several studies suggested that the dose of antigen has an effect on the immune response (e.g. Fehr and Ochsenbein, 2004; Munang'andu *et al*., 2013; Rothoeft *et al*., 2003), and might also have a significant effect on the change of oxidative status (Armour *et al*., 2020).

Second, when investigating the effects of immune response or infection on oxidative status, I found that effect sizes for markers of oxidation were significantly larger than those of non-enzymatic antioxidant or enzymatic antioxidant markers. A closer investigation into the effect of each particular assay suggests that the choice of laboratory assay might affect results of an immunostimulation experiment. Two markers of oxidative damage (protein carbonyls and TBARS) showed a strong increase in immunostimulated individuals, indicating that they might be candidate tools to assess the physiological consequences of infections or the effects of pharmacological treatments on sick animals. By contrast, there was some variation in the direction of the response among non-enzymatic and enzymatic antioxidants. These results suggest that the antioxidant response to immunostimulation might not be easily predictable without consideration of factors that might affect it, such as the individual condition or quality, the phase of the immune response, life-history stage or the quality of diet (e.g. van de Crommenacker *et al*., 2012; Garcia *et al.*, 2011; Zhao *et al*., 2019). For example, the up- or down-regulation of antioxidant enzymes might be regulated in such a manner in order to maintain an efficient immune response. The pro-oxidant hydrogen peroxide is produced by immune cells, and it can also be precursor of other compounds, such as the hypochlorous acid, which is a powerful antibacterial and antifungal pro-oxidant (Halliwell and Gutteridge, 2015; Winterbourn, 2002). Thus, a strict regulation of antioxidant enzymes, such as catalase or glutathione peroxidase that both remove hydrogen peroxide from tissues, might be important to not compromise the activity of immune cells in the initial stages of an immune response.

A third caveat of this study is that effect sizes differed among tissues, and the stronger effects were detected for gut and muscle. This result is not surprising because it is widely accepted that tissues differ in sensitivity to oxidative stress or antioxidant expression (Halliwell and Gutteridge, 2015). The adaptive meaning of this tissue effect is not understood yet. It might be that animals strategically displace oxidative costs on those tissues that have a lower impact on fitness under particular circumstances. This hypothesis has been proposed several times to explain why the effects of reproductive investment or of migratory status on the oxidative status vary among tissues (e.g. Marasco *et al*., 2021; Yang *et al*., 2013). This hypothesis has not received, however, any formal investigation so far.

Another caveat of this study is that individuals facing with an experimental immunostimulation or an infection might suppress other functions (e.g. metabolic rate, foraging activity or reproduction) that in turn affect the oxidative status, generating heterogeneity in strength and direction of effect sizes. For example, immune-challenged Egyptian fruit bats (*Rousettus aegyptiacus*) reduce significantly their foraging activity compared to control bats (Moreno *et al*., 2021). Finally, the similarity in effect sizes between correlational and experimental studies suggests that the pro-oxidants produced by parasites might have a small effect on the host oxidative status. This topic has, however, been poorly investigated so far.

Finally, this work also identified several gaps and needs in current literature. Firstly, more work is needed for amphibians and reptiles, which were very underrepresented. This is particularly relevant given the high vulnerability of these two taxa to emerging infectious diseases and their deteriorating conservation status (e.g. Ferguson *et al*., 2018; Price *et al*., 2014). Secondly, I could not test any effects of sex because most studies did not assess whether oxidative costs of immune response differ between females and males. This is unfortunate because there is variation in immune function and oxidative status, as well as life-history trade-offs between sexes (Costantini, 2018; Klein and Flanagan, 2016). Thirdly, we need more long-term longitudinal studies to identify mechanisms that determine the costs of chronic infections and of co-infections, the roles of resistance-tolerance mechanisms, the relevance of selective disappearance, and the seasonality in responses. Fourthly, we also need more experimental manipulations of key resources (e.g. food quality and quantity, competition, pollutant exposure) that might influence the relationship between immune function and oxidative status. Finally, developing models that account for effects of physiological costs on immunological performance might be interesting, illuminating and important for (i) understanding the persistence and spatial spread of infectious agents, and the host–pathogen coevolution and (ii) implementing effective conservation actions.

## Conclusions

The findings of these phylogenetic meta-analyses show congruent patterns in the link between either immune response or infection status and oxidative status across taxonomic classes. These results were consistent across experimental and correlational studies indicating that results of observational studies might suggest cause-effect relationships between infection and oxidative status. In contrast to my predictions, effect sizes were significantly larger in studies on captive animals than in those on free-living animals. Also, contrary to my predictions, oxidative costs of immune response were similar between young and adult individuals and were not associated with the species’ pace of life. These meta-analyses also show that it is very important to take into careful consideration some methodological aspects of study designs because they might affect interpretation and comparability of results and help understanding the mechanisms underlying the impacts of the type of immunostimulant and of tissue on oxidative status.

In conclusion, the outcomes of these meta-analyses show that perturbations of oxidative status are one widespread physiological effect of immune function across vertebrates that should be considered carefully when addressing the causes and consequences of variation in immunological performance in animal populations of conservation concern. Understanding physiological impacts of pathogens on organisms will be critical to assessing individual health and maintaining viable captive and wild populations.

## Data availability statement

The full dataset is provided as supplementary material.

## Supplementary Material

Web_Material_coac018
