# Supplementary material for: A meta-analysis of impacts of immune response and infection on oxidative status in vertebrates
Source: Conserv Physiol. 2022 Apr 6;10(1):coac018. doi: 10.1093/conphys/coac018 (PMC9040321; doi:10.1093/conphys/coac018)
Supplement: Web_Material_coac018 [file web_material_coac018.zip › supplementary_material.docx]

**A meta-analysis of impacts of immune response and**

**infection on oxidative status in vertebrates**

David Costantini

**Correspondence** : Unité Physiologie Moléculaire et Adaptation, UMR 7221, Muséum National d’Histoire Naturelle, CNRS, CP32, 57 rue Cuvier 75005 Paris, France

Email : [david.costantini@mnhn.fr](mailto:david.costantini@mnhn.fr) ; Telephone: 0033(0)140795374

Figure S1. PRISMA flow diagram.


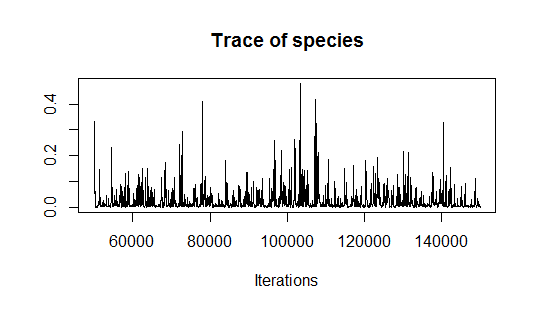


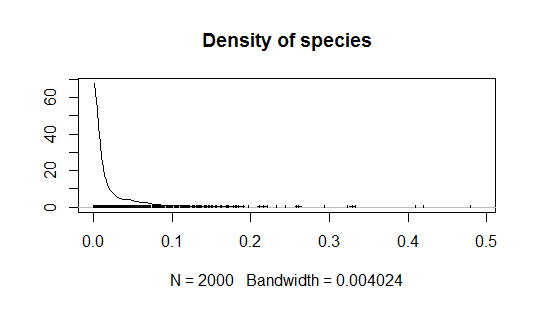


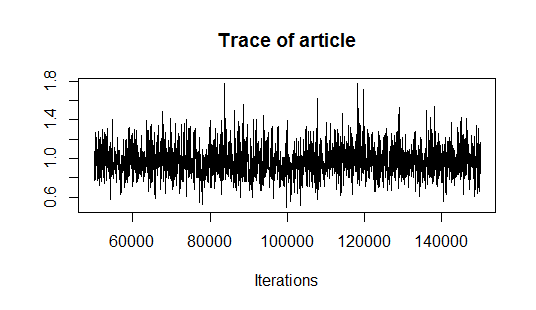


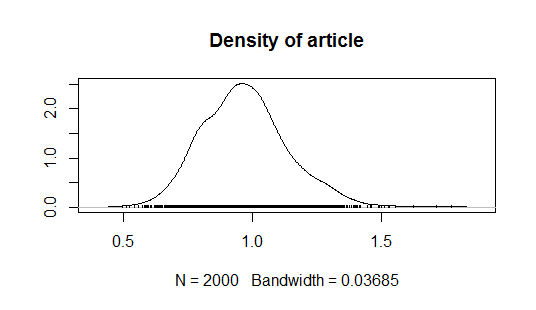


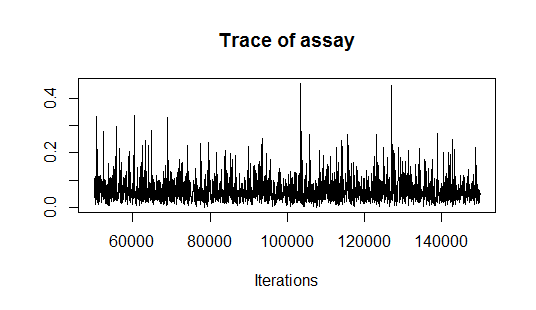


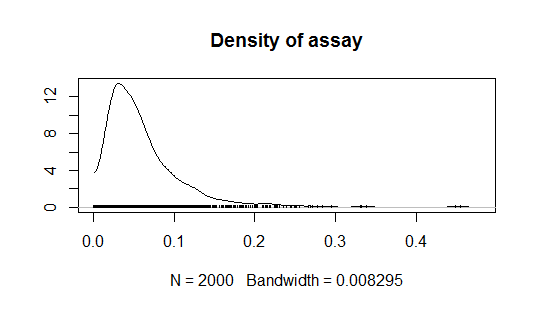


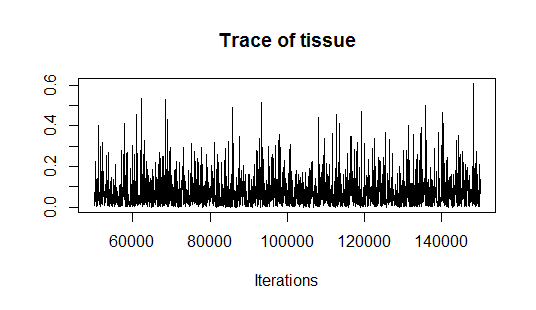


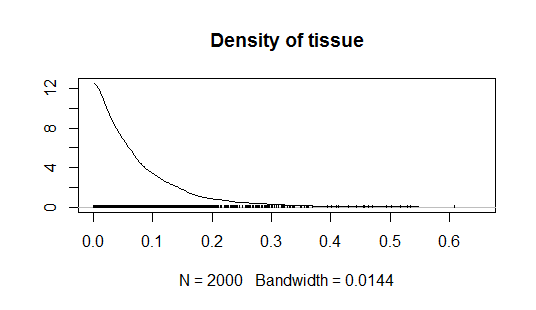


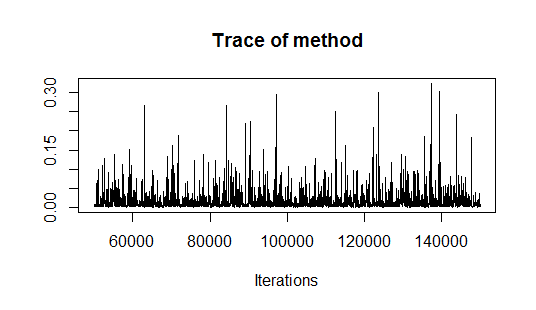


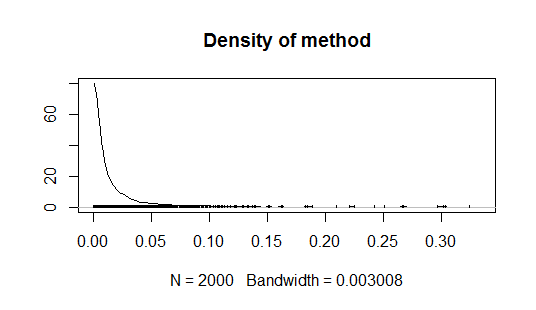


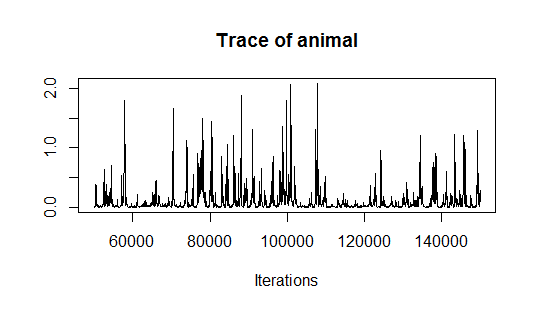


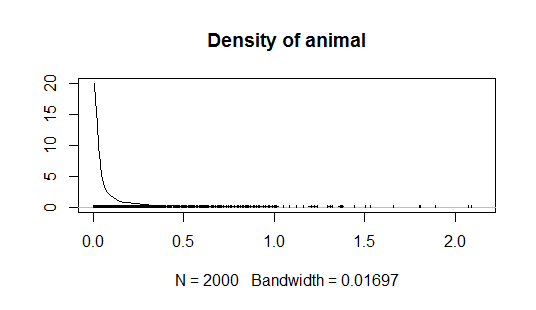


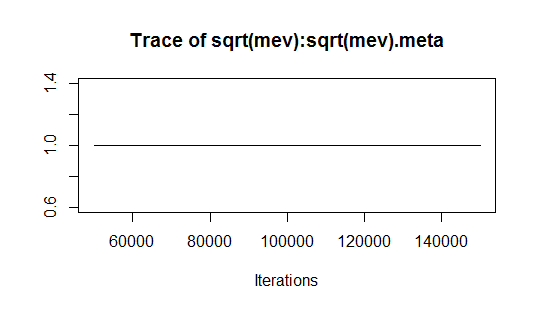

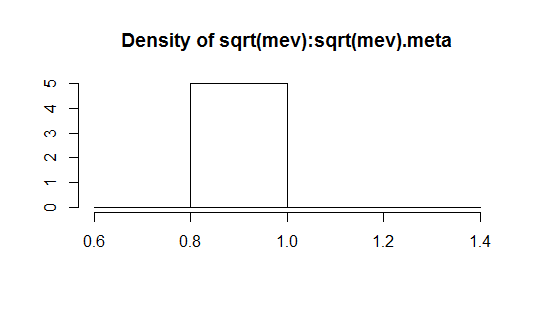


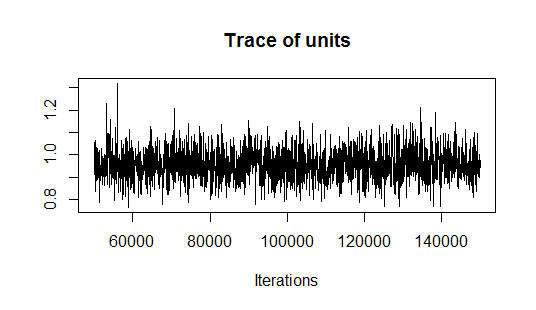


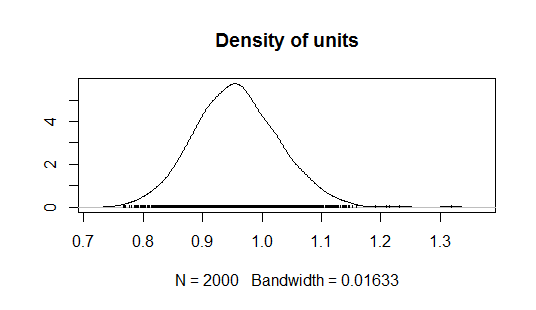


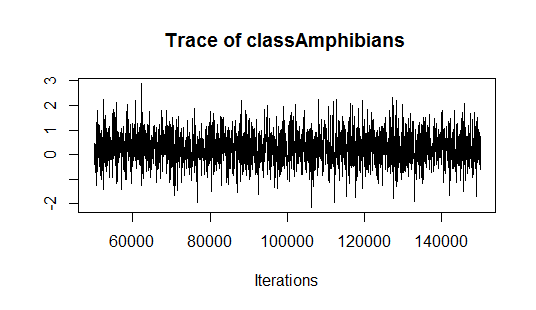


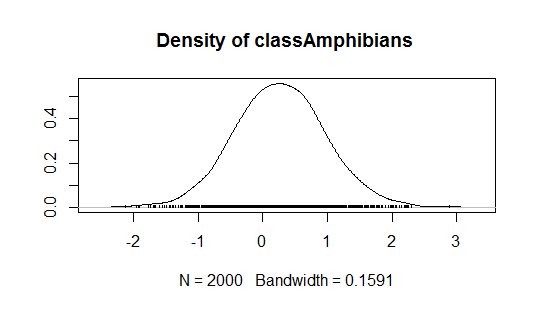


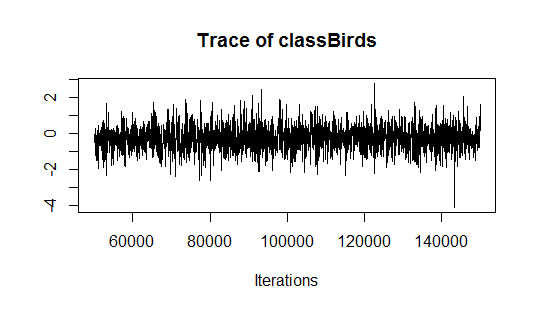


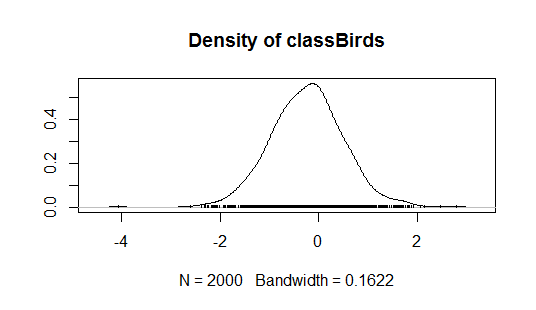


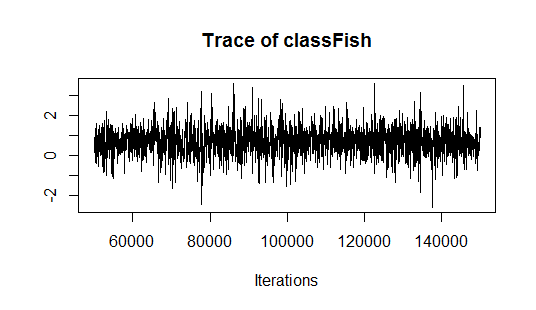


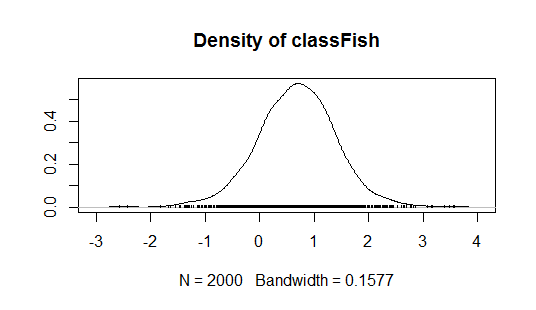


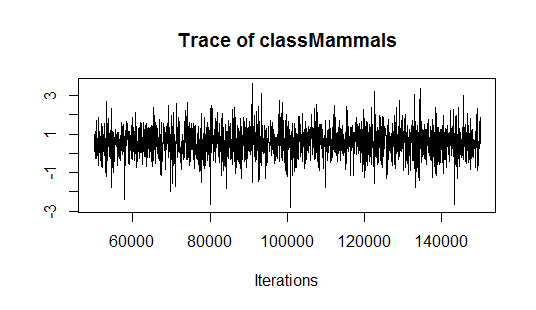


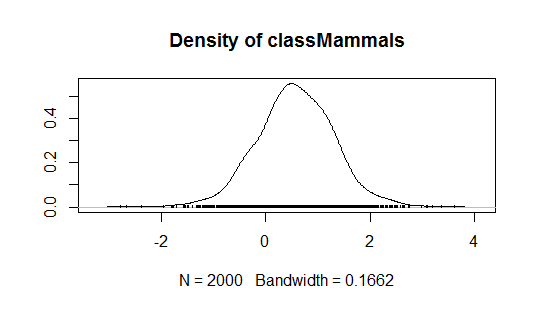


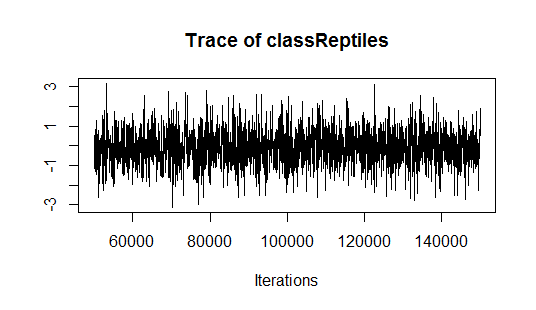


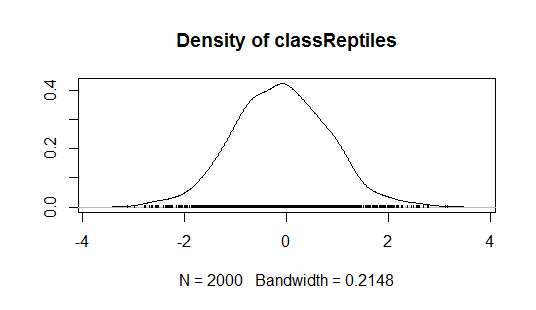


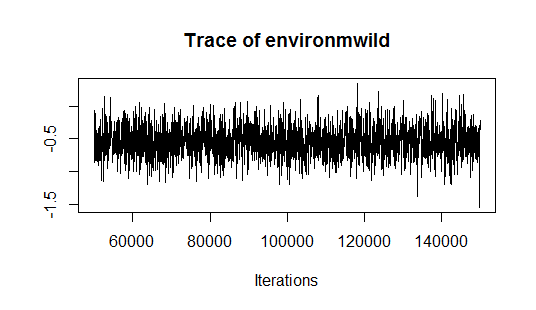


**Trace of experimental environment (wild)**


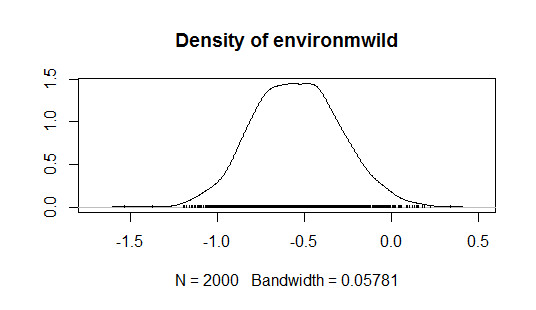


**Density of experimental environment (wild)**


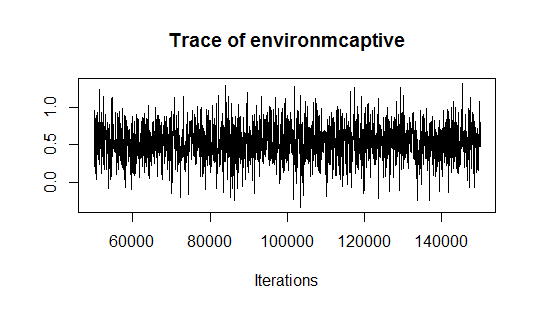


**Trace of experimental environment (captivity)**


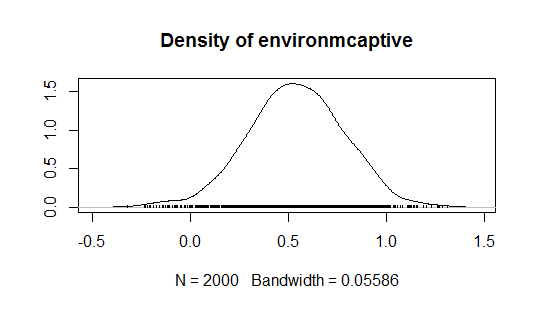


**Density of experimental environment (captivity)**


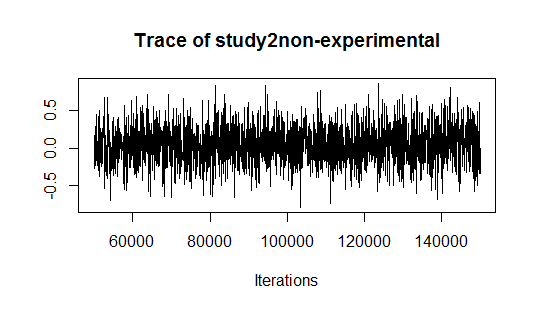


**Trace of study design (non-experimental)**


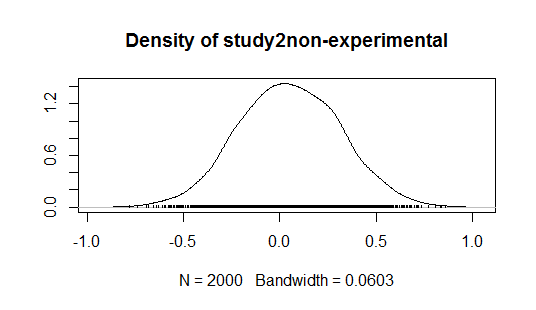


**Density of study design (non-experimental)**


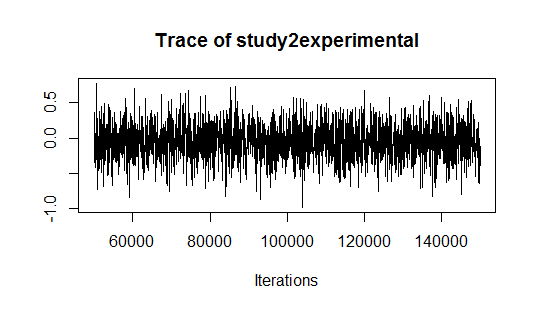


**Trace of study design (experimental)**


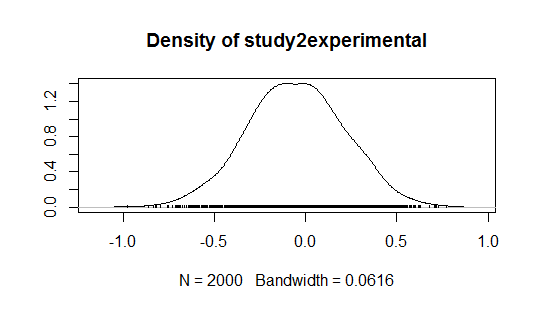


**Density of study design (experimental)**


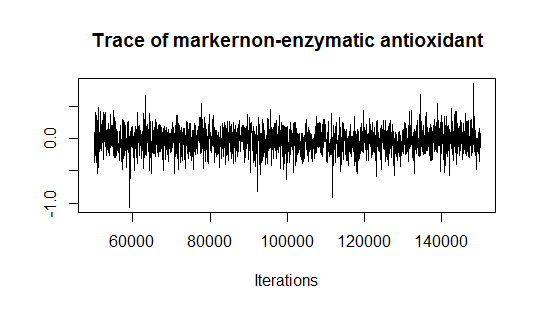


**Trace of marker category (non-enzymatic antioxidant)**


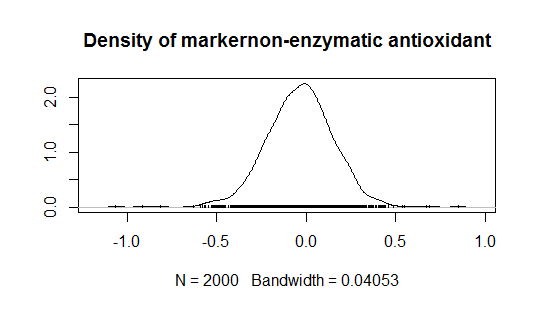


**Density of marker category (non-enzymatic antioxidant)**


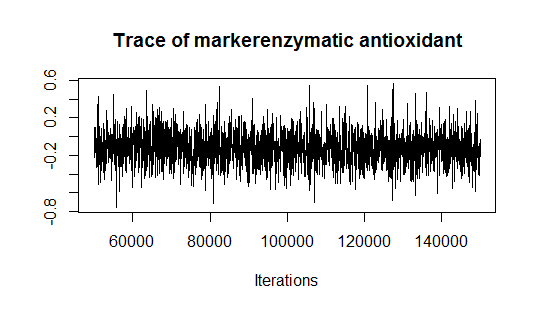


**Trace of marker category (enzymatic antioxidant)**


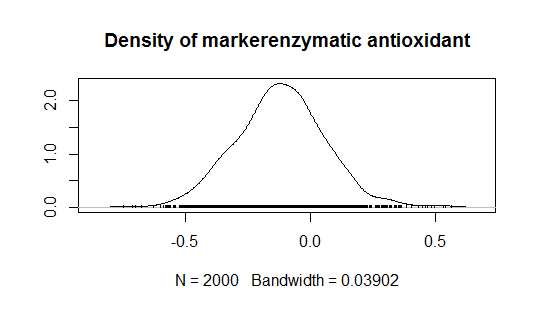


**Density of marker category (enzymatic antioxidant)**


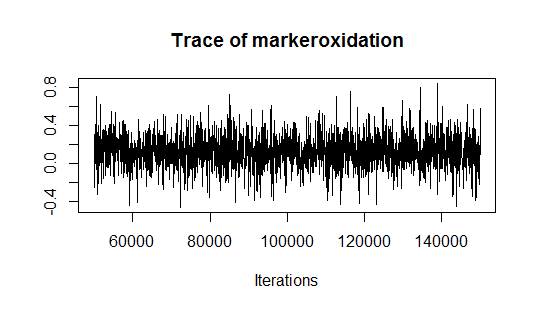


**Trace of marker category (oxidation)**


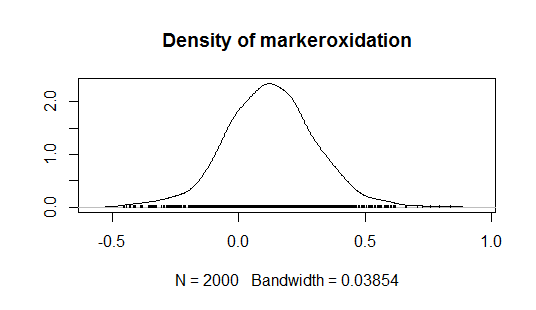


**Density of marker category (oxidation)**


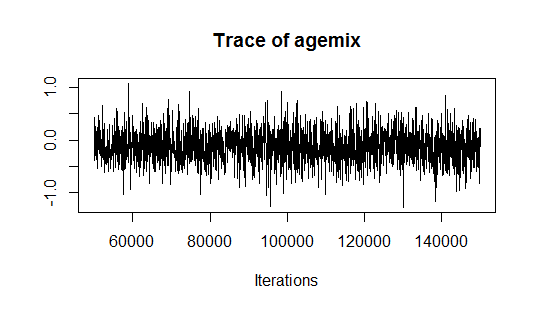


**Trace of age category (mix)**


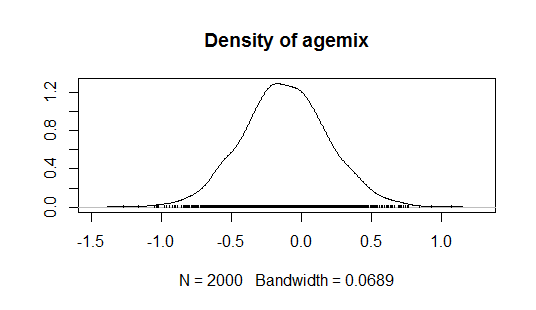


**Density of age category (mix)**


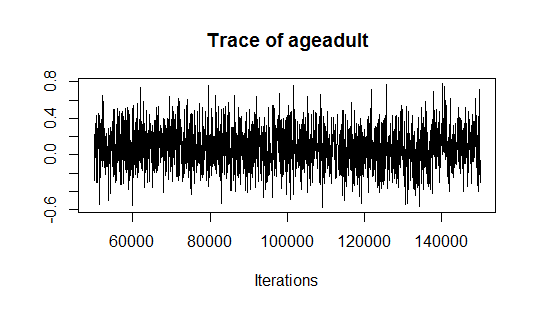


**Trace of age category (adult)**


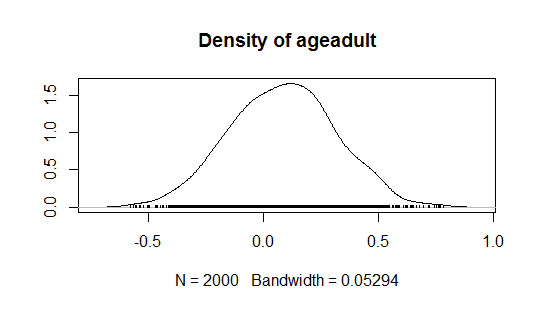


**Density of age category (adult)**


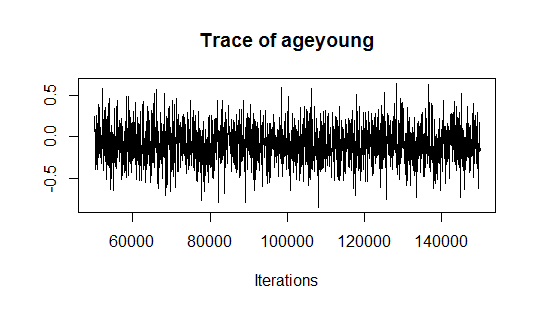


**Trace of age category (young)**


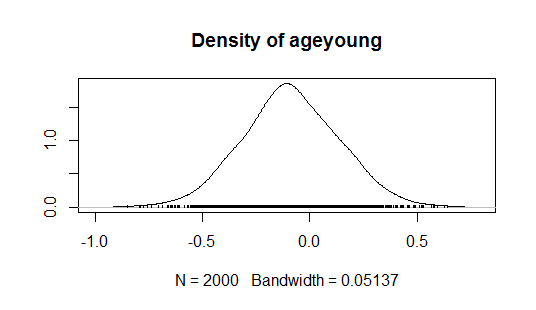


**Density of age category (young)**

Figure S2. Trace and density plots for random and fixed factors included in the full models with unsigned effect sizes. Note that the factor animal refers to the phylogeny. Estimates are shown in X-axis and Y-axis of Density and Trace plots, respectively. Y-axis label for all Density graphs is density.


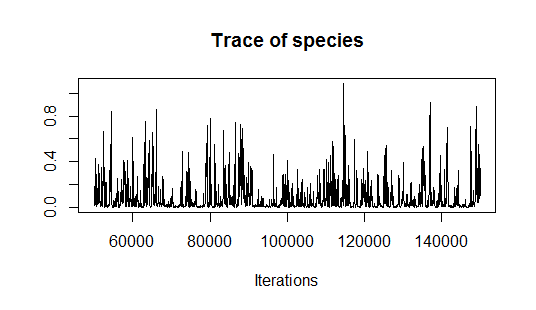


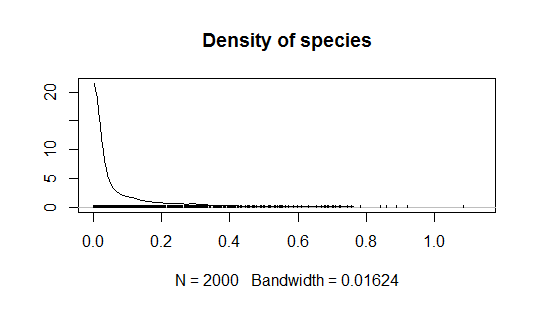


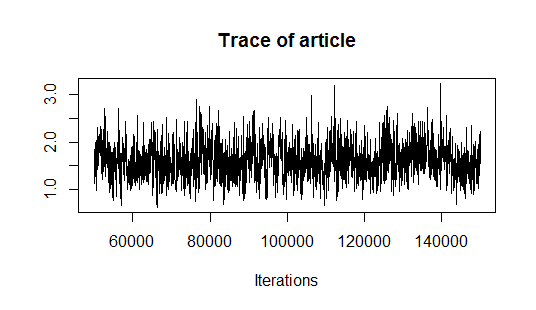


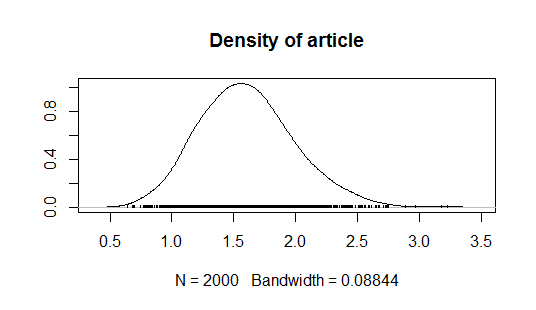


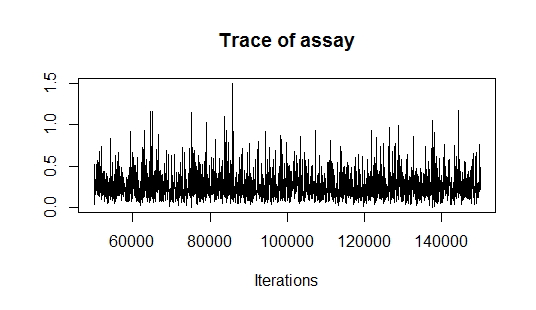


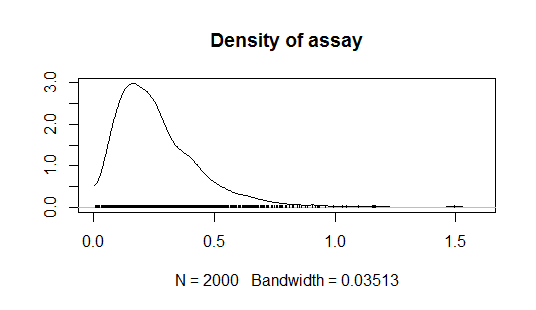


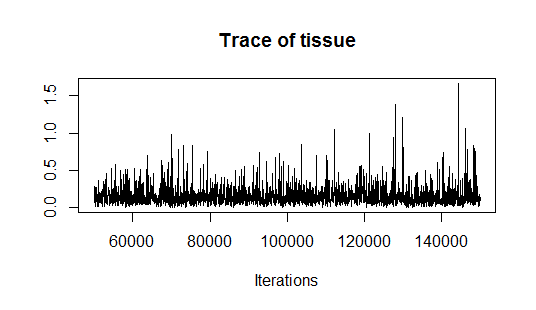


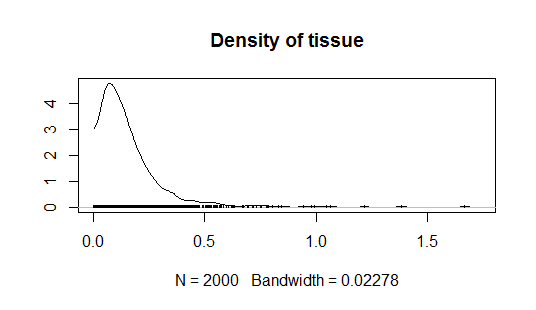


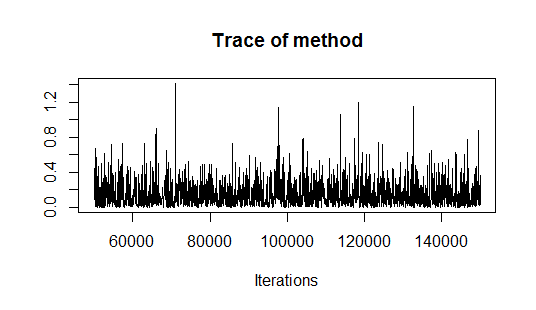


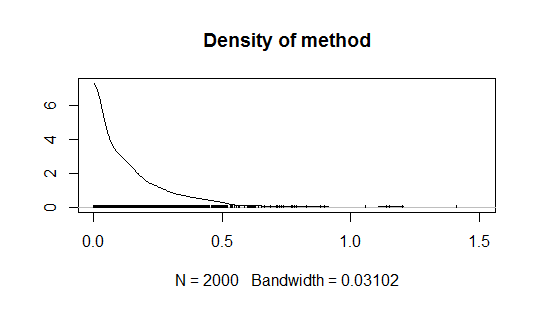


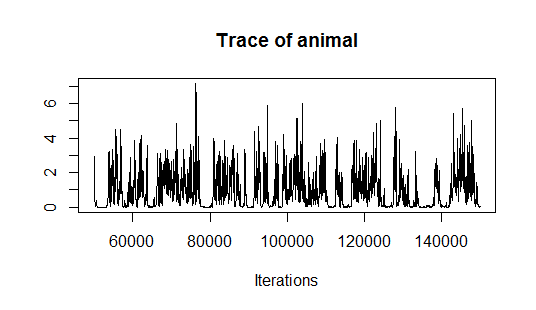


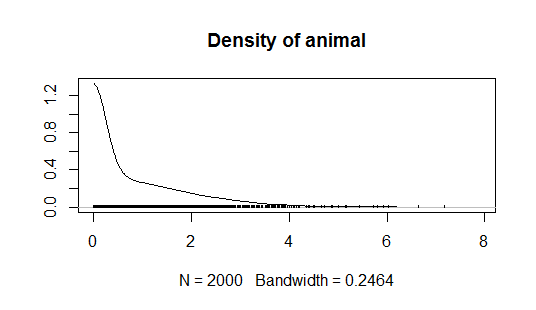


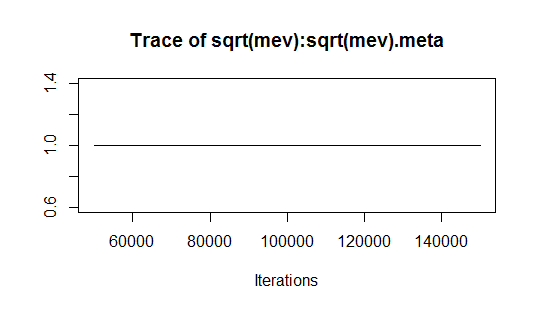


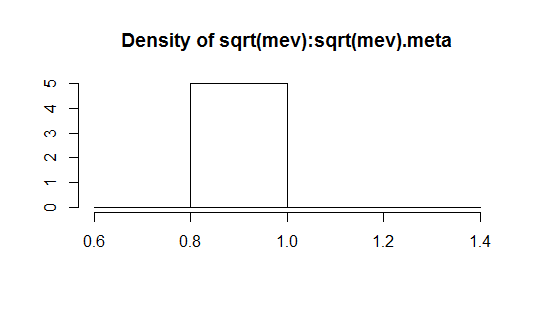


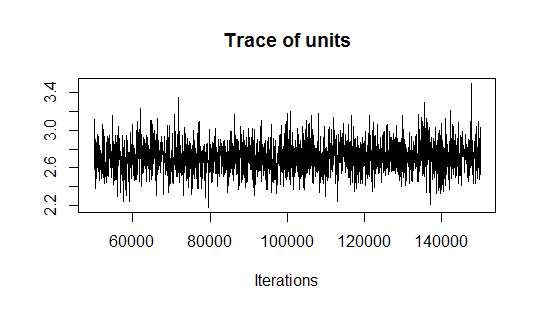


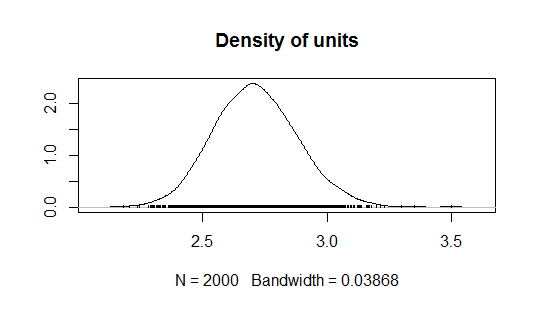


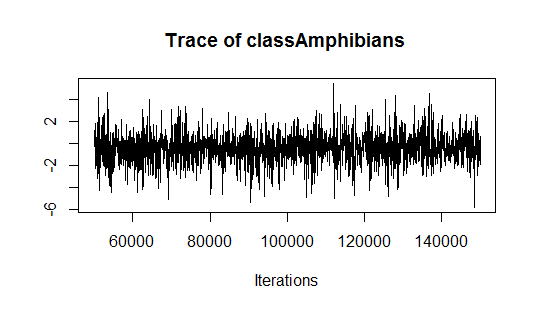


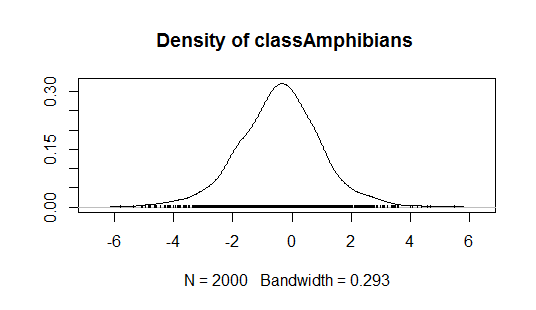


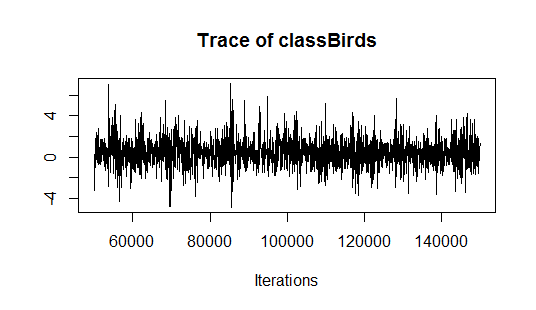


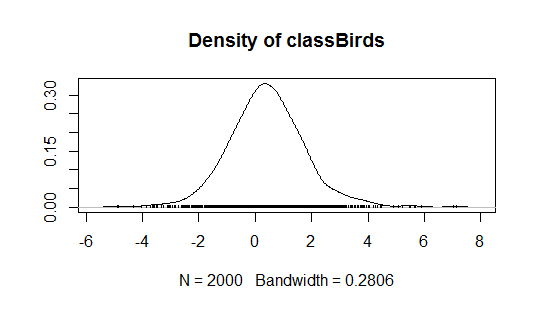


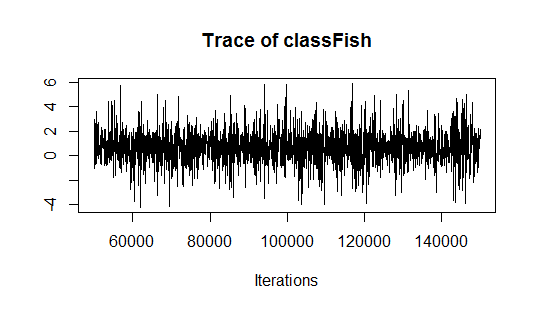


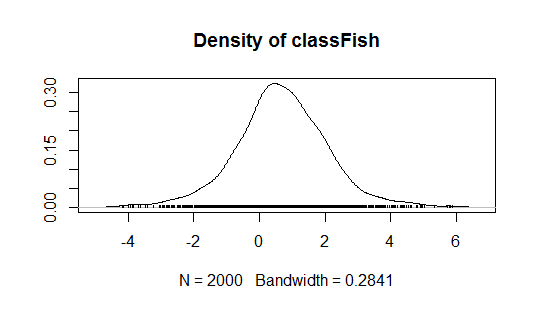


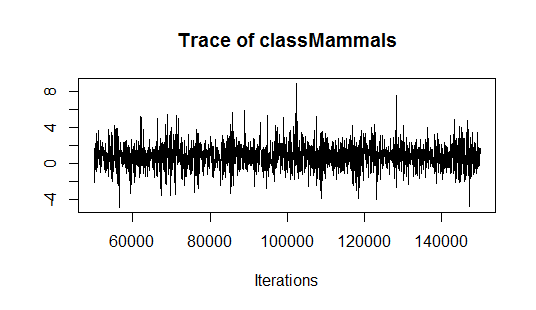


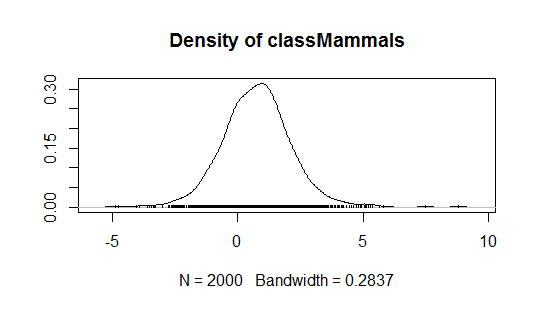


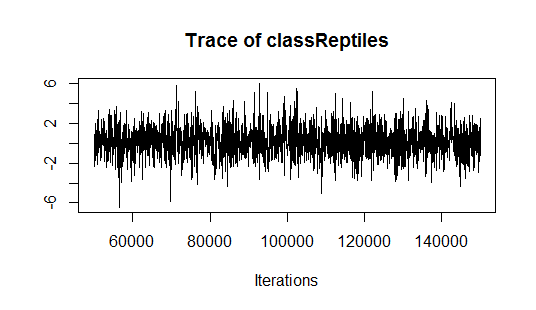


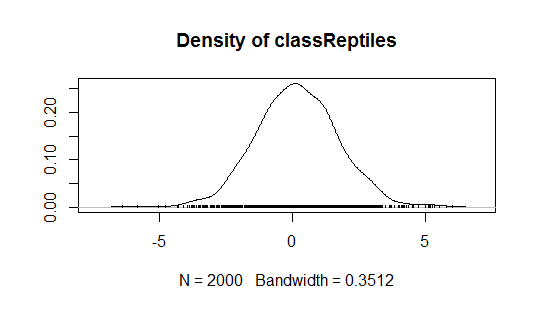


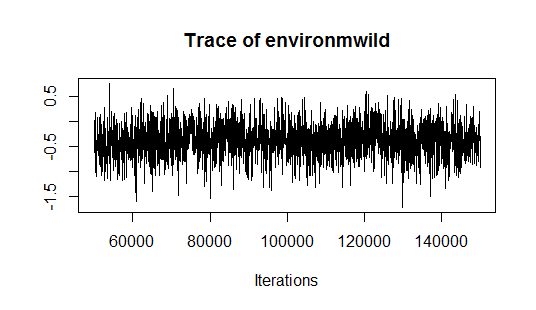


**Trace of experimental environment (wild)**


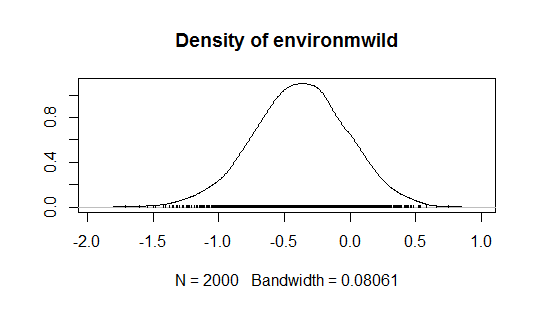


**Density of experimental environment (wild)**


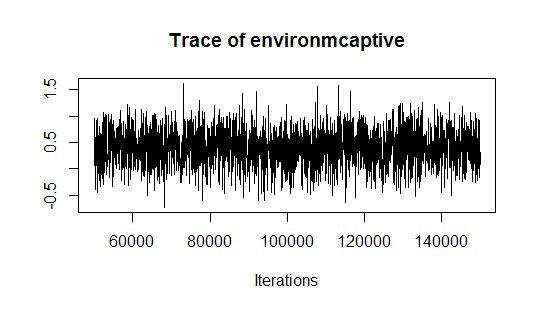


**Trace of experimental environment (captivity)**


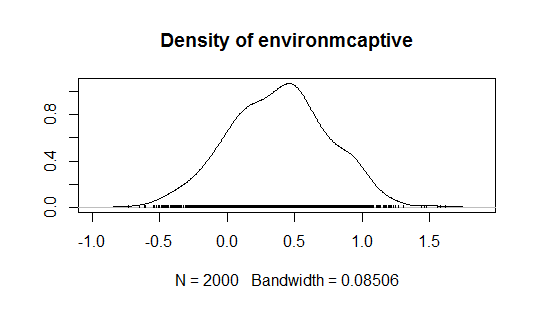


**Density of experimental environment (captivity)**


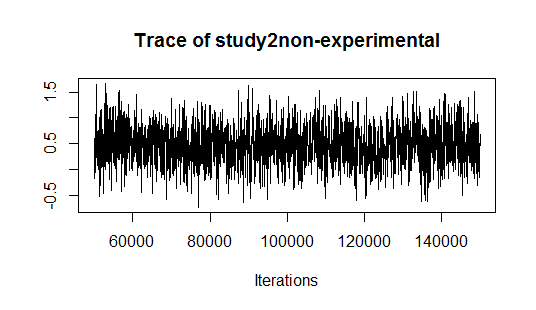


**Trace of study design (non-experimental)**


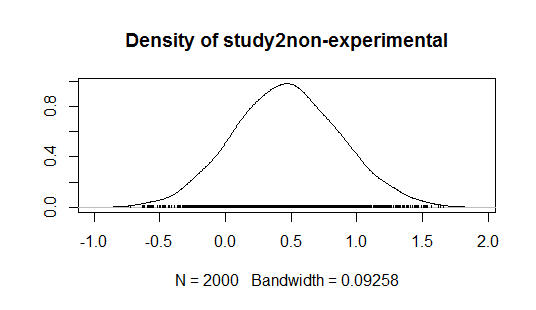


**Density of study design (non-experimental)**


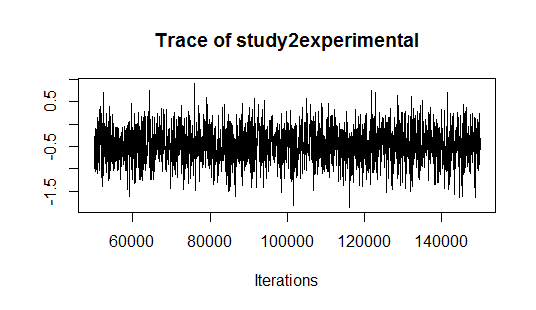


**Trace of study design (experimental)**

**Density of study design (experimental)**


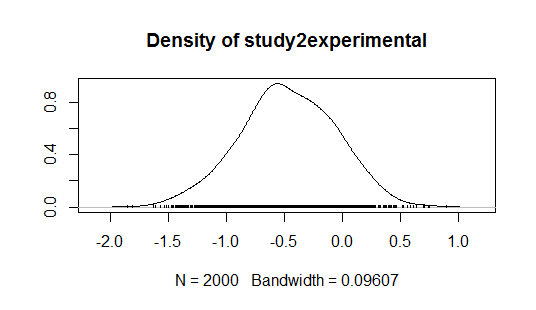


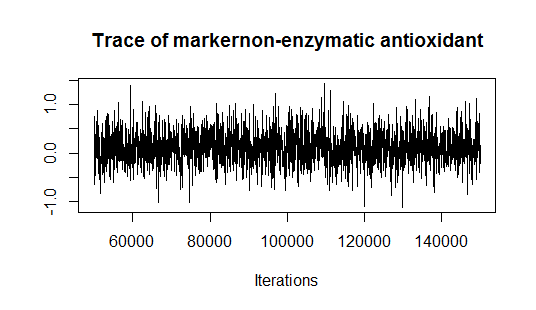


**Trace of marker category (non-enzymatic antioxidant)**


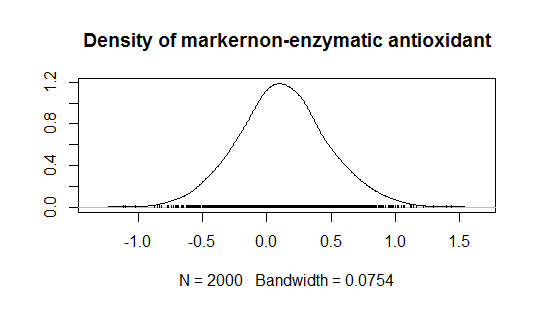


**Density of marker category (non-enzymatic antioxidant)**


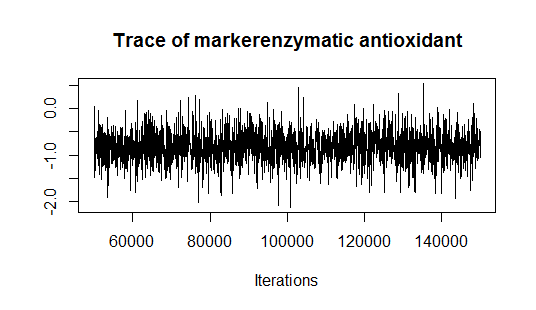


**Trace of marker category (oxidation)**

**Trace of marker category (enzymatic antioxidant)**


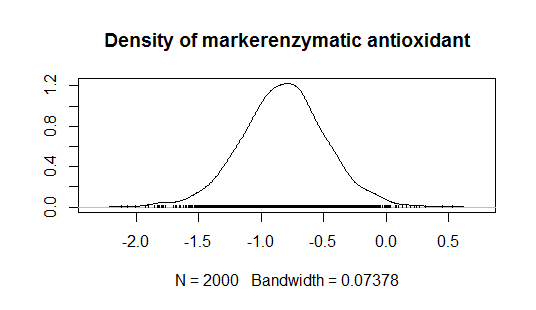


**Density of marker category (enzymatic antioxidant)**


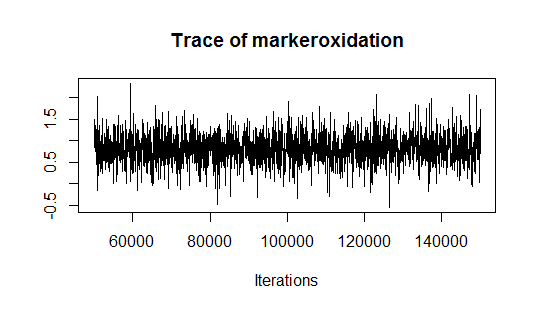


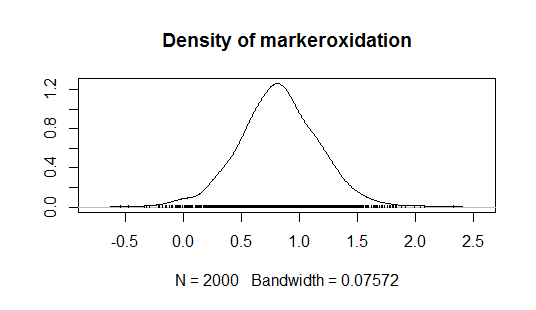


**Density of marker category (oxidation)**


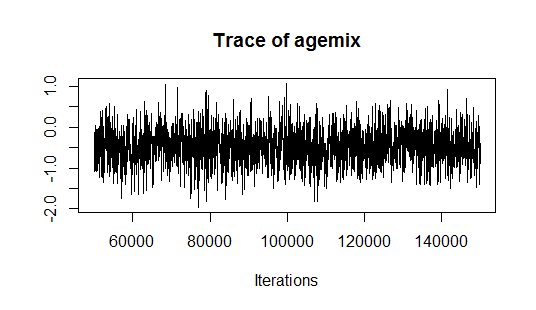


**Trace of age category (mix)**


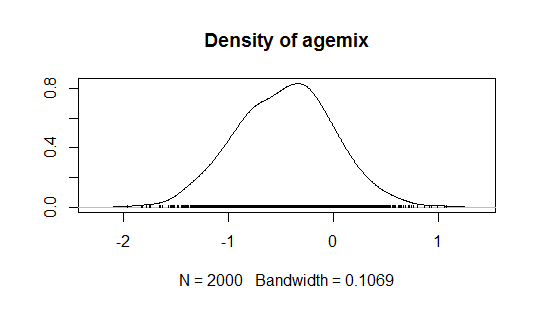


**Density of age category (mix)**


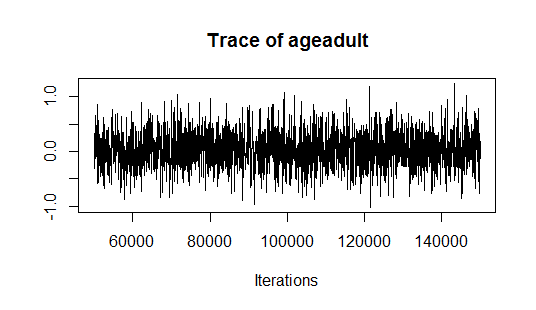


**Trace of age category (adult)**


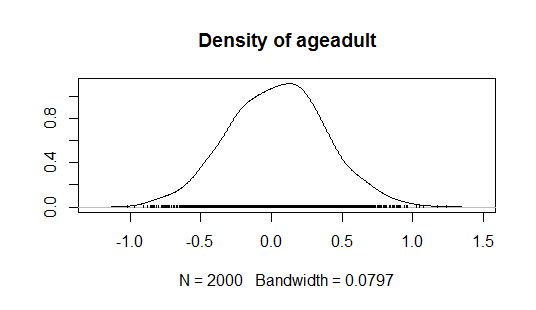


**Density of age category (adult)**


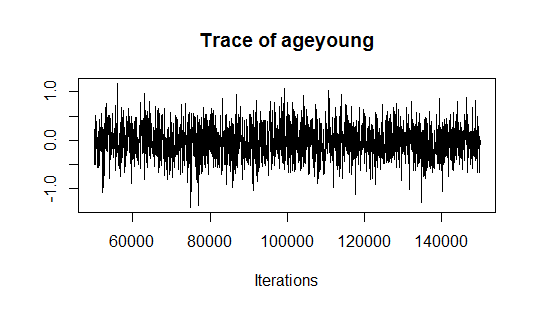


**Trace of age category (young)**


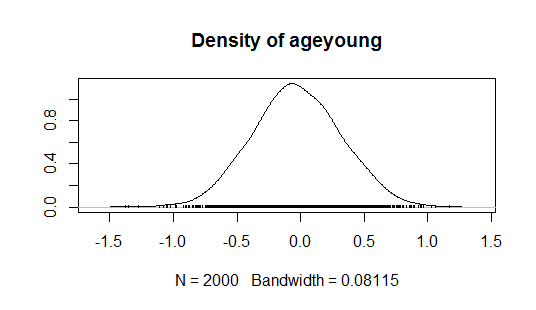


**Density of age category (young)**

Figure S3. Trace and density plots for random and fixed factors included in the full models with signed effect sizes. Note that the factor animal refers to the phylogeny. Estimates are shown in X-axis and Y-axis of Density and Trace plots, respectively. Y-axis label for all Density graphs is density.

Figure S4. Funnel plot illustrating the relationship between effect size (n = 1262) expressed as standardized Hedges’g (obtained using the compute.es package in R) and log-transformed sample size derived from each article included in the meta-analysis.

| blood |
| --- |
| brain |
| central nervous system |
| eye |
| gills |
| gonads |
| gut |
| heart |
| kidney |
| liver |
| lungs |
| muscle |
| skin |
| spleen |
| swim bladder |
| urine |
| whole-body |

Table S1. List of tissues and of other biological matrices included in the meta-analyses.

| **Category** | **Pathogen** | **Taxonomic class** |
| --- | --- | --- |
| Bacterial infection | Aeromonas hydrophila | Fish |
| Bacterial infection | Aeromonas schubertii | Fish |
| Bacterial infection | Aeromonas sorbia | Fish |
| Bacterial infection | Edwardsiella tarda | Fish |
| Bacterial infection | Enterococcus faecalis | Fish |
| Bacterial infection | Escherichia coli | Fish |
| Bacterial infection | Isospora | Birds |
| Bacterial infection | Leptospira interrogans | Mammals |
| Bacterial infection | Mycobacterium bovis | Mammals |
| Bacterial infection | Mycoplasma gallisepticum | Birds |
| Bacterial infection | Photobacterium damselae | Fish |
| Bacterial infection | Proteus vulgaris | Fish |
| Bacterial infection | Pseudomonas aeruginosa | Fish |
| Bacterial infection | Streptococcus iniae | Fish |
| Bacterial infection | Vibrio fischeri | Fish |
| Bacterial infection | Yersinia ruckeri | Fish |
| Fungal infection | Aeromonas hydrophila | Fish |
| Fungal infection | Aphanomyces laevis | Fish |
| Fungal infection | Phoma herbarum | Fish |
| Fungal infection | Pseudogymnoascus destructans | Mammals |
| Fungal infection | Saprolegnia | Amphibians |
| Fungal infection | Saprolegnia | Fish |
| Fungal infection | Saprolegnia ferax | Fish |
| Protozoan infection | Cryptocaryon irritans | Fish |
| Protozoan infection | Haemoproteus columbae | Birds |
| Protozoan infection | Ichthyophthirius multifiliis | Fish |
| Protozoan infection | Neospora caninum | Mammals |
| Protozoan infection | Neospora caninum | Mammals |
| Protozoan infection | Philasterides dicentrarchi | Fish |
| Protozoan infection | Plasmodium | Birds |
| Protozoan infection | Plasmodium gonderi | Mammals |
| Protozoan infection | Plasmodium knowlesi | Mammals |
| Protozoan infection | Plasmodium mandrilli | Mammals |
| Protozoan infection | Plasmodium relictum | Birds |
| Protozoan infection | Plasmodium+Haemoproteus | Birds |
| Protozoan infection | Toxoplasma gondii | Mammals |
| Protozoan infection | Trypanosoma evansi | Mammals |
| Viral infection | Avipoxvirus | Birds |
| Viral infection | grass carp hemorrhagic virus | Fish |
| Viral infection | Herpesvirus | Birds |
| Viral infection | Herpesvirus | Mammals |
| Viral infection | Herpesvirus | Reptiles |
| Viral infection | Myxoma virus+Rabbit haemorrhagic disease virus | Mammals |
| Viral infection | Myxoma virus (MV) | Mammals |
| Viral infection | Rabbit haemorrhagic disease virus (RHDV) | Mammals |
| Worm infection | Amplicaecum africanum | Amphibians |
| Worm infection | Anguillicola crassus | Fish |
| Worm infection | Apophallus brevis | Fish |
| Worm infection | Clinostomum complanatum | Fish |
| Worm infection | Contracaecum sp. | Fish |
| Worm infection | Cryptocotyle | Fish |
| Worm infection | Echinococcus granulosus | Mammals |
| Worm infection | Echinostoma sp. | Amphibians |
| Worm infection | Gigantobilharzia huronensis | Birds |
| Worm infection | Gorgoderidae | Amphibians |
| Worm infection | Heterakis gallinarum | Birds |
| Worm infection | Hysterothylacium aduncum | Fish |
| Worm infection | Opisthorchis viverrini | Mammals |
| Worm infection | Ornithodiplostomum ptychocheilus | Fish |
| Worm infection | Oswaldocruzia sp. | Amphibians |
| Worm infection | Polymorphus sp. | Fish |
| Worm infection | Pomphorhynchus sp. | Fish |
| Worm infection | Ptychobothrium sp. | Fish |
| Worm infection | Raphidascaris acus | Fish |
| Worm infection | Rhabdias sp. | Amphibians |
| Worm infection | Spiroxys sp. | Fish |
| Worm infection | Thaparocleidus sp. | Fish |
| Worm infection | Trichostrongylus tenuis | Birds |
| Worm infection | Uvulifer ambloplitis | Fish |

Table S2. List of parasites included in the meta-analyses.

| Effects | Posterior mean | Lower 95% CI | Upper 95% CI | pMCMC |
| --- | --- | --- | --- | --- |
| *Unsigned effect sizes* |  |  |  |  |
| Species (random factor) | 0.023 | 0.0001 | 0.096 |  |
| Article (random factor) | 0.971 | 0.668 | 1.313 |  |
| Laboratory assay (random factor) | 0.061 | 0.001 | 0.150 |  |
| Tissue (random factor) | 0.075 | 0.0003 | 0.234 |  |
| Type of immunostimulant (random factor) | 0.018 | 0.0002 | 0.077 |  |
| Phylogeny (random factor) | 0.111 | 0.0003 | 0.560 |  |
| Amphibians vs. Birds | -0.238 | -1.804 | 1.097 | 0.734 |
| Amphibians vs. Fish | 0.683 | -0.718 | 2.155 | 0.312 |
| Amphibians vs. Mammals | 0.567 | -0.881 | 2.059 | 0.410 |
| Amphibians vs. Reptiles | -0.104 | -2.071 | 1.603 | 0.890 |
| Birds vs. Fish | 0.897 | 0.001 | 1.890 | 0.064 |
| Birds vs. Mammals | 0.819 | -0.011 | 1.713 | 0.057 |
| Birds vs. Reptiles | 0.147 | -1.219 | 1.565 | 0.851 |
| Mammals vs. Fish | 0.108 | -0.913 | 0.984 | 0.760 |
| Mammals vs. Reptiles | -0.679 | -2.221 | 0.586 | 0.352 |
| Fish vs. Reptiles | -0.800 | -2.213 | 0.718 | 0.277 |
| Captive vs. Wild | -0.544 | -1.003 | -0.032 | 0.029 |
| Experimental vs. Non-experimental | 0.051 | -0.436 | 0.565 | 0.865 |
| Enzymatic antioxidant vs. Non-enzymatic antioxidant | -0.033 | -0.398 | 0.301 | 0.867 |
| Enzymatic antioxidant vs. Oxidation marker | 0.127 | -0.245 | 0.465 | 0.460 |
| Oxidation marker vs. Non-enzymatic antioxidant | -0.154 | -0.522 | 0.168 | 0.368 |
| Adult vs. Mix | -0.118 | -0.683 | 0.529 | 0.697 |
| Adult vs. Young | -0.089 | -0.526 | 0.357 | 0.666 |
| Young vs. Mix | -0.020 | -0.590 | 0.543 | 0.945 |
| *Signed effect sizes* |  |  |  |  |
| Species (random factor) | 0.082 | 0.0002 | 0.354 |  |
| Article (random factor) | 1.604 | 0.927 | 2.414 |  |
| Laboratory assay (random factor) | 0.267 | 0.019 | 0.600 |  |
| Tissue (random factor) | 0.155 | 0.0005 | 0.416 |  |
| Type of immunostimulant (random factor) | 0.144 | 0.0003 | 0.441 |  |
| Phylogeny (random factor) | 0.922 | 0.0003 | 3.091 |  |
| Amphibians vs. Birds | 0.417 | -2.220 | 3.169 | 0.737 |
| Amphibians vs. Fish | 0.678 | -1.960 | 3.759 | 0.566 |
| Amphibians vs. Mammals | 0.759 | -2.053 | 3.378 | 0.544 |
| Amphibians vs. Reptiles | 0.178 | -2.560 | 3.332 | 0.907 |
| Birds vs. Fish | 0.303 | -2.407 | 2.710 | 0.696 |
| Birds vs. Mammals | 0.303 | -1.959 | 2.835 | 0.690 |
| Birds vs. Reptiles | -0.184 | -2.596 | 2.208 | 0.893 |
| Mammals vs. Fish | -0.046 | -2.731 | 2.792 | 0.970 |
| Mammals vs. Reptiles | -0.474 | -3.424 | 2.063 | 0.755 |
| Fish vs. Reptiles | -0.474 | -3.370 | 2.503 | 0.750 |
| Captive vs. Wild | -0.381 | -1.080 | 0.309 | 0.286 |
| Experimental vs. Non-experimental | 0.469 | -0.281 | 1.268 | 0.248 |
| Enzymatic antioxidant vs. Non-enzymatic antioxidant | 0.123 | -0.560 | 0.827 | 0.721 |
| Enzymatic antioxidant vs. Oxidation marker | 0.818 | 0.189 | 1.578 | 0.028 |
| Oxidation marker vs. Non-enzymatic antioxidant | -0.702 | -1.258 | -0.050 | 0.014 |
| Adult vs. Mix | -0.465 | -1.411 | 0.372 | 0.297 |
| Adult vs. Young | -0.030 | -0.703 | 0.641 | 0.908 |
| Young vs. Mix | -0.439 | -1.274 | 0.353 | 0.280 |

Table S3. Results of the full dataset; n = 1262.

| Effects | Posterior mean | Lower 95% CI | Upper 95% CI | pMCMC |
| --- | --- | --- | --- | --- |
| *Unsigned effect sizes* |  |  |  |  |
| Species (random factor) | 0.024 | 0.0004 | 0.100 |  |
| Article (random factor) | 0.956 | 0.722 | 1.339 |  |
| Laboratory assay (random factor) | 0.067 | 0.005 | 0.155 |  |
| Tissue (random factor) | 0.081 | 0.0003 | 0.257 |  |
| Type of immunostimulant (random factor) | 0.018 | 0.0002 | 0.075 |  |
| Phylogeny (random factor) | 0.135 | 0.0002 | 0.614 |  |
| Amphibians vs. Birds | 0.059 | -1.651 | 1.658 | 0.949 |
| Amphibians vs. Fish | 1.168 | -0.343 | 2.873 | 0.138 |
| Amphibians vs. Mammals | 0.900 | -0.768 | 2.534 | 0.272 |
| Amphibians vs. Reptiles | 0.176 | -1.784 | 2.134 | 0.852 |
| Birds vs. Fish | 1.143 | 0.086 | 2.032 | 0.031 |
| Birds vs. Mammals | 0.858 | -0.222 | 1.708 | 0.076 |
| Birds vs. Reptiles | 0.141 | -1.257 | 1.638 | 0.851 |
| Mammals vs. Fish | 0.293 | -0.667 | 1.288 | 0.491 |
| Mammals vs. Reptiles | -0.695 | -2.174 | 0.791 | 0.326 |
| Fish vs. Reptiles | -0.960 | -2.439 | 0.563 | 0.216 |
| Captive vs. Wild | -0.568 | -1.030 | -0.084 | 0.017 |
| Experimental vs. Non-experimental | 0.032 | -0.507 | 0.518 | 0.889 |
| Enzymatic antioxidant vs. Non-enzymatic antioxidant | 0.031 | -0.385 | 0.386 | 0.816 |
| Enzymatic antioxidant vs. Oxidation marker | 0.186 | -0.185 | 0.606 | 0.325 |
| Oxidation marker vs. Non-enzymatic antioxidant | -0.151 | -0.510 | 0.211 | 0.413 |
| Adult vs. Mix | -0.231 | -0.885 | 0.478 | 0.501 |
| Adult vs. Young | -0.089 | -0.568 | 0.381 | 0.730 |
| Young vs. Mix | -0.158 | -0.816 | 0.444 | 0.622 |
| *Signed effect sizes* |  |  |  |  |
| Species (random factor) | 0.071 | 0.0003 | 0.342 |  |
| Article (random factor) | 1.260 | 0.646 | 2.039 |  |
| Laboratory assay (random factor) | 0.298 | 0.051 | 0.677 |  |
| Tissue (random factor) | 0.249 | 0.0008 | 0.613 |  |
| Type of immunostimulant (random factor) | 0.180 | 0.0004 | 0.554 |  |
| Phylogeny (random factor) | 2.281 | 0.0004 | 4.835 |  |
| Amphibians vs. Birds | 0.554 | -3.030 | 4.778 | 0.772 |
| Amphibians vs. Fish | 0.910 | -2.837 | 5.141 | 0.619 |
| Amphibians vs. Mammals | 0.794 | -3.249 | 4.510 | 0.680 |
| Amphibians vs. Reptiles | 0.333 | -3.512 | 4.021 | 0.894 |
| Birds vs. Fish | 0.292 | -3.631 | 3.831 | 0.791 |
| Birds vs. Mammals | 0.248 | -2.829 | 3.792 | 0.831 |
| Birds vs. Reptiles | -0.221 | -3.135 | 2.810 | 0.895 |
| Mammals vs. Fish | 0.121 | -3.796 | 3.649 | 0.895 |
| Mammals vs. Reptiles | -0.410 | -3.649 | 2.893 | 0.777 |
| Fish vs. Reptiles | -0.509 | -4.418 | 2.957 | 0.768 |
| Captive vs. Wild | -0.359 | -1.097 | 0.326 | 0.340 |
| Experimental vs. Non-experimental | 0.354 | -0.450 | 1.160 | 0.385 |
| Enzymatic antioxidant vs. Non-enzymatic antioxidant | 0.129 | -0.604 | 0.843 | 0.702 |
| Enzymatic antioxidant vs. Oxidation marker | 0.946 | 0.213 | 1.628 | 0.013 |
| Oxidation marker vs. Non-enzymatic antioxidant | -0.817 | -1.427 | -0.148 | 0.016 |
| Adult vs. Mix | -0.637 | -1.654 | 0.286 | 0.197 |
| Adult vs. Young | -0.079 | -0.741 | 0.600 | 0.823 |
| Young vs. Mix | -0.531 | -1.503 | 0.315 | 0.244 |

Table S4. Results of the full dataset after the exclusion of eight species that were replaced with closely-related species in order to build the tree.

| Effects | Posterior mean | Lower 95% CI | Upper 95% CI | pMCMC |
| --- | --- | --- | --- | --- |
| *Unsigned effect sizes* |  |  |  |  |
| Species (random factor) | 0.021 | 0.0002 | 0.086 |  |
| Article (random factor) | 0.750 | 0.476 | 1.016 |  |
| Laboratory assay (random factor) | 0.053 | 0.0030 | 0.129 |  |
| Tissue (random factor) | 0.027 | 0.0002 | 0.099 |  |
| Type of immunostimulant (random factor) | 0.016 | 0.0002 | 0.058 |  |
| Phylogeny (random factor) | 0.082 | 0.0002 | 0.378 |  |
| Amphibians vs. Birds | -0.298 | -1.528 | 0.858 | 0.619 |
| Amphibians vs. Fish | 0.560 | -0.698 | 1.715 | 0.353 |
| Amphibians vs. Mammals | 0.468 | -0.722 | 1.746 | 0.462 |
| Amphibians vs. Reptiles | -0.180 | -1.743 | 1.357 | 0.852 |
| Birds vs. Fish | 0.847 | -0.290 | 1.561 | 0.063 |
| Birds vs. Mammals | 0.752 | -0.055 | 1.618 | 0.078 |
| Birds vs. Reptiles | 0.139 | -1.129 | 1.418 | 0.841 |
| Mammals vs. Fish | 0.084 | -0.831 | 0.949 | 0.825 |
| Mammals vs. Reptiles | -0.653 | -2.045 | 0.613 | 0.331 |
| Fish vs. Reptiles | -0.722 | -1.748 | 0.616 | 0.269 |
| Captive vs. Wild | -0.505 | -0.919 | -0.073 | 0.022 |
| Experimental vs. Non-experimental | 0.071 | -0.370 | 0.537 | 0.762 |
| Enzymatic antioxidant vs. Non-enzymatic antioxidant | -0.107 | -0.452 | 0.200 | 0.532 |
| Enzymatic antioxidant vs. Oxidation marker | 0.059 | -0.257 | 0.374 | 0.699 |
| Oxidation marker vs. Non-enzymatic antioxidant | -0.176 | -0.512 | 0.101 | 0.249 |
| Adult vs. Mix | -0.068 | -0.635 | 0.471 | 0.820 |
| Adult vs. Young | -0.123 | -0.524 | 0.226 | 0.542 |
| Young vs. Mix | 0.066 | -0.443 | 0.607 | 0.805 |
| *Signed effect sizes* |  |  |  |  |
| Species (random factor) | 0.072 | 0.0002 | 0.331 |  |
| Article (random factor) | 1.187 | 0.590 | 1.757 |  |
| Laboratory assay (random factor) | 0.145 | 0.0020 | 0.336 |  |
| Tissue (random factor) | 0.093 | 0.0004 | 0.266 |  |
| Type of immunostimulant (random factor) | 0.111 | 0.0003 | 0.363 |  |
| Phylogeny (random factor) | 0.832 | 0.0005 | 2.536 |  |
| Amphibians vs. Birds | 0.394 | -2.197 | 2.908 | 0.735 |
| Amphibians vs. Fish | 0.585 | -1.982 | 3.094 | 0.604 |
| Amphibians vs. Mammals | 0.635 | -1.861 | 3.241 | 0.595 |
| Amphibians vs. Reptiles | 0.215 | -2.405 | 3.172 | 0.901 |
| Birds vs. Fish | 0.188 | -2.371 | 2.600 | 0.804 |
| Birds vs. Mammals | 0.182 | -2.106 | 2.402 | 0.792 |
| Birds vs. Reptiles | -0.186 | -2.352 | 2.013 | 0.851 |
| Mammals vs. Fish | -0.042 | -2.254 | 2.487 | 0.936 |
| Mammals vs. Reptiles | -0.420 | -2.762 | 2.108 | 0.719 |
| Fish vs. Reptiles | -0.345 | -3.031 | 2.262 | 0.740 |
| Captive vs. Wild | -0.354 | -0.954 | 0.282 | 0.253 |
| Experimental vs. Non-experimental | 0.429 | -0.292 | 1.103 | 0.246 |
| Enzymatic antioxidant vs. Non-enzymatic antioxidant | -0.013 | -0.542 | 0.539 | 0.973 |
| Enzymatic antioxidant vs. Oxidation marker | 0.736 | 0.202 | 1.243 | 0.008 |
| Oxidation marker vs. Non-enzymatic antioxidant | -0.738 | -1.222 | -0.260 | 0.006 |
| Adult vs. Mix | -0.413 | -1.206 | 0.363 | 0.308 |
| Adult vs. Young | -0.069 | -0.656 | 0.527 | 0.814 |
| Young vs. Mix | -0.344 | -1.085 | 0.376 | 0.372 |

Table S5. Results of the full dataset (n = 1214) after the exclusion of very large effect sizes (>10, n = 48).

| Effects | Posterior mean | Lower 95% CI | Upper 95% CI | pMCMC |
| --- | --- | --- | --- | --- |
| Antigen injection (unsigned effect size) |  |  |  |  |
| with centered covariate | -0.042 | -0.146 | 0.065 | 0.450 |
| without centered covariate | -0.0001 | -0.0004 | 0.0002 | 0.443 |
|  |  |  |  |  |
| Antigen injection (signed effect size) |  |  |  |  |
| with centered covariate | -0.044 | -0.206 | 0.127 | 0.610 |
| without centered covariate | -0.0001 | -0.0006 | 0.0003 | 0.625 |
|  |  |  |  |  |
| Exposure to a living parasite (unsigned effect size) |  |  |  |  |
| with centered covariate | -0.171 | -0.390 | 0.059 | 0.132 |
| without centered covariate | -0.0002 | -0.0005 | 0.00006 | 0.139 |
|  |  |  |  |  |
| Exposure to a living parasite (signed effect size) |  |  |  |  |
| with centered covariate | 0.101 | -0.238 | 0.472 | 0.612 |
| without centered covariate | 0.0001 | -0.0003 | 0.0006 | 0.604 |

Table S6. Results about the effects of the time elapsed from antigen injection to final sampling (n = 158) or of the time elapsed since the start of the infection with a living parasite until the final sampling (n = 518).

| Effects | Posterior mean | Lower 95% CI | Upper 95% CI | pMCMC |
| --- | --- | --- | --- | --- |
| *Unsigned effect sizes* |  |  |  |  |
| Bacterial infection vs. Fungal infection | -0.025 | -0.944 | 0.840 | 0.953 |
| Bacterial infection vs. LPS E. coli | 0.293 | -0.575 | 1.105 | 0.512 |
| Bacterial infection vs. Protozoan infection | -0.016 | -0.874 | 0.774 | 0.984 |
| Bacterial infection vs. Viral infection | -0.201 | -1.329 | 0.897 | 0.746 |
| Bacterial infection vs. Worm infection | -0.565 | -1.331 | 0.172 | 0.133 |
| Fungal infection vs. LPS E. coli | 0.302 | -0.800 | 1.356 | 0.599 |
| Fungal infection vs. Protozoan infection | 0.006 | -1.043 | 1.072 | 0.977 |
| Fungal infection vs. Viral infection | -0.175 | -1.525 | 1.045 | 0.798 |
| Fungal infection vs. Worm infection | -0.546 | -1.519 | 0.433 | 0.288 |
| LPS E. coli vs. Protozoan infection | -0.319 | -1.264 | 0.487 | 0.460 |
| LPS E. coli vs. Viral infection | -0.491 | -1.603 | 0.683 | 0.418 |
| LPS E. coli vs. Worm infection | -0.866 | -1.735 | -0.010 | 0.048 |
| Protozoan infection vs. Viral infection | -0.193 | -1.282 | 0.879 | 0.746 |
| Protozoan infection vs. Worm infection | -0.532 | -1.411 | 0.345 | 0.238 |
| Viral infection vs. Worm infection | -0.357 | -1.473 | 0.866 | 0.548 |
| *Signed effect sizes* |  |  |  |  |
| Bacterial infection vs. Fungal infection | 1.227 | -0.027 | 2.334 | 0.043 |
| Bacterial infection vs. LPS E. coli | 1.960 | 0.901 | 3.151 | <0.001 |
| Bacterial infection vs. Protozoan infection | 0.861 | -0.244 | 1.848 | 0.121 |
| Bacterial infection vs. Viral infection | 1.116 | -0.346 | 2.506 | 0.131 |
| Bacterial infection vs. Worm infection | 0.051 | -0.861 | 1.127 | 0.927 |
| Fungal infection vs. LPS E. coli | 0.734 | -0.674 | 2.131 | 0.317 |
| Fungal infection vs. Protozoan infection | -0.365 | -1.776 | 1.113 | 0.595 |
| Fungal infection vs. Viral infection | -0.135 | -1.967 | 1.514 | 0.876 |
| Fungal infection vs. Worm infection | -1.163 | -2.480 | 0.135 | 0.086 |
| LPS E. coli vs. Protozoan infection | -1.109 | -2.285 | 0.128 | 0.075 |
| LPS E. coli vs. Viral infection | -0.870 | -2.551 | 0.599 | 0.270 |
| LPS E. coli vs. Worm infection | -1.891 | -3.187 | -0.712 | 0.005 |
| Protozoan infection vs. Viral infection | 0.234 | -1.257 | 1.688 | 0.780 |
| Protozoan infection vs. Worm infection | -0.829 | -1.955 | 0.388 | 0.181 |
| Viral infection vs. Worm infection | -1.032 | -2.499 | 0.461 | 0.178 |

Table S7. Contrasts among immunostimulant types (n = 1033). Each category includes studies that tested the effect of a given immunostimulant, e.g. bacterial infection refers to studies that tested the effects of various species of bacteria on oxidative status markers.

| Effects | Posterior mean | Lower 95% CI | Upper 95% CI | pMCMC |
| --- | --- | --- | --- | --- |
| *Unsigned effect sizes* |  |  |  |  |
| CAT vs. dROMs | -0.298 | -0.925 | 0.340 | 0.348 |
| CAT vs. GPx | 0.304 | -0.014 | 0.620 | 0.062 |
| CAT vs. GR | -0.199 | -0.526 | 0.140 | 0.245 |
| CAT vs. GSH | 0.106 | -0.205 | 0.430 | 0.526 |
| CAT vs. GST | 0.458 | 0.150 | 0.797 | 0.002 |
| CAT vs. OXY | -0.426 | -1.086 | 0.150 | 0.171 |
| CAT vs. Protein carbonyls | 0.681 | 0.214 | 1.202 | 0.007 |
| CAT vs. SOD | -0.010 | -0.294 | 0.245 | 0.952 |
| CAT vs. TBARS | 0.005 | -0.256 | 0.267 | 0.977 |
| dROMs vs. GPx | 0.617 | -0.039 | 1.248 | 0.067 |
| dROMs vs. GR | 0.113 | -0.505 | 0.808 | 0.699 |
| dROMs vs. GSH | 0.419 | -0.220 | 1.071 | 0.222 |
| dROMs vs. GST | 0.775 | 0.163 | 1.535 | 0.028 |
| dROMs vs. OXY | -0.130 | -0.510 | 0.325 | 0.551 |
| dROMs vs. Protein carbonyls | 1.009 | 0.321 | 1.740 | 0.003 |
| dROMs vs. SOD | 0.301 | -0.329 | 0.945 | 0.351 |
| dROMs vs. TBARS | 0.310 | -0.318 | 0.906 | 0.333 |
| GPx vs. GR | -0.506 | -0.856 | -0.126 | 0.004 |
| GPx vs. GSH | -0.193 | -0.547 | 0.172 | 0.294 |
| GPx vs. GST | 0.156 | -0.215 | 0.547 | 0.433 |
| GPx vs. OXY | -0.729 | -1.417 | -0.077 | 0.035 |
| GPx vs. Protein carbonyls | 0.383 | -0.105 | 0.851 | 0.117 |
| GPx vs. SOD | -0.320 | -0.634 | -0.015 | 0.047 |
| GPx vs. TBARS | -0.303 | -0.627 | -0.001 | 0.060 |
| GSH vs. GR | -0.313 | -0.694 | 0.092 | 0.110 |
| GSH vs. GST | 0.355 | -0.005 | 0.747 | 0.069 |
| GSH vs. OXY | -0.549 | -1.216 | 0.073 | 0.096 |
| GSH vs. Protein carbonyls | 0.575 | 0.069 | 1.073 | 0.033 |
| GSH vs. SOD | -0.115 | -0.426 | 0.206 | 0.480 |
| GSH vs. TBARS | -0.110 | -0.450 | 0.173 | 0.504 |
| GST vs. GR | -0.652 | -1.039 | -0.281 | 0.001 |
| GST vs. OXY | -0.906 | -1.581 | -0.241 | 0.006 |
| GST vs. Protein carbonyls | 0.218 | -0.285 | 0.764 | 0.438 |
| GST vs. SOD | -0.462 | -0.799 | -0.124 | 0.008 |
| GST vs. TBARS | -0.456 | -0.769 | -0.131 | 0.008 |
| OXY vs. GR | 0.244 | -0.449 | 0.902 | 0.473 |
| OXY vs. Protein carbonyls | 1.117 | 0.439 | 1.830 | 0.004 |
| OXY vs. SOD | 0.429 | -0.186 | 1.091 | 0.190 |
| OXY vs. TBARS | 0.440 | -0.190 | 1.089 | 0.175 |
| Protein carbonyls vs. GR | -0.876 | -1.427 | -0.380 | 0.001 |
| Protein carbonyls vs. SOD | -0.686 | -1.188 | -0.206 | 0.006 |
| Protein carbonyls vs. TBARS | -0.675 | -1.189 | -0.200 | 0.007 |
| SOD vs. GR | -0.190 | -0.534 | 0.165 | 0.279 |
| SOD vs. TBARS | 0.009 | -0.256 | 0.277 | 0.944 |
| TBARS vs. GR | -0.200 | -0.534 | 0.138 | 0.239 |
| *Signed effect sizes* |  |  |  |  |
| CAT vs. dROMs | -0.121 | -1.071 | 0.800 | 0.813 |
| CAT vs. GPx | -0.013 | -0.498 | 0.459 | 0.94 |
| CAT vs. GR | -0.480 | -1.038 | 0.017 | 0.082 |
| CAT vs. GSH | -0.410 | -0.904 | 0.099 | 0.112 |
| CAT vs. GST | -1.102 | -1.556 | -0.558 | <0.001 |
| CAT vs. OXY | -0.556 | -1.489 | 0.448 | 0.263 |
| CAT vs. Protein carbonyls | 1.669 | 0.908 | 2.434 | <0.001 |
| CAT vs. SOD | -0.186 | -0.582 | 0.238 | 0.377 |
| CAT vs. TBARS | 0.522 | 0.103 | 0.897 | 0.006 |
| dROMs vs. GPx | 0.116 | -0.809 | 1.062 | 0.800 |
| dROMs vs. GR | -0.358 | -1.347 | 0.576 | 0.472 |
| dROMs vs. GSH | -0.292 | -1.231 | 0.584 | 0.550 |
| dROMs vs. GST | -0.992 | -2.005 | -0.080 | 0.046 |
| dROMs vs. OXY | -0.436 | -1.135 | 0.288 | 0.224 |
| dROMs vs. Protein carbonyls | 1.790 | 0.714 | 2.853 | <0.001 |
| dROMs vs. SOD | -0.069 | -0.932 | 0.872 | 0.894 |
| dROMs vs. TBARS | 0.640 | -0.255 | 1.507 | 0.177 |
| GPx vs. GR | -0.476 | -1.033 | 0.128 | 0.108 |
| GPx vs. GSH | -0.401 | -0.970 | 0.164 | 0.178 |
| GPx vs. GST | -1.103 | -1.686 | -0.500 | <0.001 |
| GPx vs. OXY | -0.522 | -1.412 | 0.507 | 0.295 |
| GPx vs. Protein carbonyls | 1.683 | 0.980 | 2.509 | <0.001 |
| GPx vs. SOD | -0.178 | -0.629 | 0.320 | 0.474 |
| GPx vs. TBARS | 0.527 | 0.055 | 1.027 | 0.033 |
| GSH vs. GR | -0.068 | -0.641 | 0.549 | 0.814 |
| GSH vs. GST | -0.704 | -1.348 | -0.166 | 0.019 |
| GSH vs. OXY | -0.168 | -1.070 | 0.832 | 0.741 |
| GSH vs. Protein carbonyls | 2.063 | 1.312 | 2.857 | <0.001 |
| GSH vs. SOD | 0.216 | -0.297 | 0.721 | 0.404 |
| GSH vs. TBARS | 0.924 | 0.444 | 1.414 | <0.001 |
| GST vs. GR | 0.618 | -0.001 | 1.237 | 0.055 |
| GST vs. OXY | 0.549 | -0.419 | 1.545 | 0.284 |
| GST vs. Protein carbonyls | 2.777 | 1.957 | 3.537 | <0.001 |
| GST vs. SOD | 0.925 | 0.366 | 1.450 | <0.001 |
| GST vs. TBARS | 1.624 | 1.070 | 2.119 | <0.001 |
| OXY vs. GR | 0.064 | -0.914 | 1.010 | 0.885 |
| OXY vs. Protein carbonyls | 2.208 | 1.005 | 3.193 | <0.001 |
| OXY vs. SOD | 0.361 | -0.546 | 1.309 | 0.424 |
| OXY vs. TBARS | 1.066 | 0.138 | 1.941 | 0.025 |
| Protein carbonyls vs. GR | -2.156 | -3.003 | -1.399 | <0.001 |
| Protein carbonyls vs. SOD | -1.868 | -2.673 | -1.156 | <0.001 |
| Protein carbonyls vs. TBARS | -1.158 | -1.852 | -0.446 | 0.002 |
| SOD vs. GR | -0.296 | -0.818 | 0.228 | 0.272 |
| SOD vs. TBARS | 0.706 | 0.279 | 1.081 | <0.001 |
| TBARS vs. GR | -0.997 | -1.560 | -0.494 | <0.001 |

Table S8. Contrasts among laboratory assays (n = 1093). Abbreviations are explained in the main text.

| Effects | Posterior mean | Lower 95% CI | Upper 95% CI | pMCMC |
| --- | --- | --- | --- | --- |
| *Unsigned effect sizes* |  |  |  |  |
| Blood vs. Gills | 0.333 | -0.066 | 0.707 | 0.102 |
| Blood vs. Gut | 1.415 | 0.769 | 2.097 | <0.001 |
| Blood vs. Kidney | -0.122 | -0.640 | 0.386 | 0.661 |
| Blood vs. Liver | 0.371 | 0.046 | 0.726 | 0.032 |
| Blood vs. Muscle | 0.440 | -0.004 | 0.886 | 0.053 |
| Blood vs. Spleen | 0.145 | -0.431 | 0.753 | 0.637 |
| Gills vs. Gut | 1.071 | 0.444 | 1.767 | 0.002 |
| Gills vs. Kidney | -0.461 | -0.986 | 0.056 | 0.083 |
| Gills vs. Liver | 0.041 | -0.284 | 0.368 | 0.823 |
| Gills vs. Muscle | 0.103 | -0.262 | 0.505 | 0.602 |
| Gills vs. Spleen | -0.188 | -0.831 | 0.431 | 0.568 |
| Gut vs. Kidney | -1.538 | -2.345 | -0.760 | <0.001 |
| Gut vs. Liver | -1.037 | -1.692 | -0.312 | 0.004 |
| Gut vs. Muscle | -0.973 | -1.740 | -0.189 | 0.017 |
| Gut vs. Spleen | -1.270 | -2.108 | -0.446 | 0.006 |
| Kidney vs. Liver | 0.504 | 0.021 | 0.951 | 0.039 |
| Kidney vs. Muscle | 0.563 | -0.011 | 1.119 | 0.052 |
| Kidney vs. Spleen | 0.287 | -0.392 | 0.934 | 0.405 |
| Liver vs. Muscle | 0.057 | -0.328 | 0.437 | 0.752 |
| Liver vs. Spleen | -0.228 | -0.778 | 0.351 | 0.426 |
| Muscle vs. Spleen | -0.301 | -0.966 | 0.364 | 0.371 |
| *Signed effect sizes* |  |  |  |  |
| Blood vs. Gills | -0.082 | -0.563 | 0.538 | 0.775 |
| Blood vs. Gut | 0.882 | -0.101 | 1.821 | 0.076 |
| Blood vs. Kidney | -0.094 | -0.839 | 0.680 | 0.821 |
| Blood vs. Liver | -0.065 | -0.514 | 0.427 | 0.799 |
| Blood vs. Muscle | 0.951 | 0.317 | 1.606 | 0.002 |
| Blood vs. Spleen | 0.508 | -0.409 | 1.298 | 0.257 |
| Gills vs. Gut | 0.952 | 0.008 | 1.886 | 0.049 |
| Gills vs. Kidney | -0.033 | -0.825 | 0.695 | 0.933 |
| Gills vs. Liver | 0.014 | -0.440 | 0.499 | 0.952 |
| Gills vs. Muscle | 1.033 | 0.472 | 1.622 | <0.001 |
| Gills vs. Spleen | 0.578 | -0.365 | 1.446 | 0.217 |
| Gut vs. Kidney | -0.983 | -2.137 | 0.077 | 0.087 |
| Gut vs. Liver | -0.928 | -1.855 | -0.001 | 0.044 |
| Gut vs. Muscle | 0.093 | -0.934 | 1.068 | 0.866 |
| Gut vs. Spleen | -0.376 | -1.466 | 0.804 | 0.527 |
| Kidney vs. Liver | 0.028 | -0.664 | 0.771 | 0.942 |
| Kidney vs. Muscle | 1.047 | 0.215 | 1.914 | 0.020 |
| Kidney vs. Spleen | 0.592 | -0.424 | 1.534 | 0.254 |
| Liver vs. Muscle | 1.010 | 0.483 | 1.569 | 0.001 |
| Liver vs. Spleen | 0.576 | -0.192 | 1.457 | 0.173 |
| Muscle vs. Spleen | -0.460 | -1.378 | 0.585 | 0.359 |

Table S9. Contrasts among tissues (n = 1183).

| Effects | Posterior mean | Lower 95% CI | Upper 95% CI | pMCMC |
| --- | --- | --- | --- | --- |
| BIRDS |  |  |  |  |
| *Unsigned effect sizes* |  |  |  |  |
| Clutch size | 0.016 | -0.031 | 0.060 | 0.469 |
| Body mass | -0.00004 | -0.0002 | 0.00006 | 0.433 |
| Captive vs. Wild | 0.069 | -0.249 | 0.344 | 0.620 |
| Experimental vs. Non-experimental | 0.042 | -0.231 | 0.334 | 0.779 |
| Enzymatic antioxidant vs. Non-enzymatic antioxidant | 0.132 | -0.054 | 0.324 | 0.172 |
| Enzymatic antioxidant vs. Oxidation marker | 0.196 | 0.0003 | 0.375 | 0.033 |
| Oxidation marker vs. Non-enzymatic antioxidant | -0.066 | -0.216 | 0.067 | 0.050 |
| Adult vs. Young | -0.088 | -0.307 | 0.137 | 0.412 |
| *Signed effect sizes* |  |  |  |  |
| Clutch size | 0.034 | -0.017 | 0.084 | 0.188 |
| Body mass | 0.00004 | -0.0001 | 0.0002 | 0.594 |
| Captive vs. Wild | 0.071 | -0.296 | 0.441 | 0.704 |
| Experimental vs. Non-experimental | -0.072 | -0.475 | 0.321 | 0.701 |
| Enzymatic antioxidant vs. Non-enzymatic antioxidant | 0.147 | -0.259 | 0.523 | 0.445 |
| Enzymatic antioxidant vs. Oxidation marker | 0.298 | -0.069 | 0.677 | 0.124 |
| Oxidation marker vs. Non-enzymatic antioxidant | -0.150 | -0.470 | 0.166 | 0.351 |
| Adult vs. Young | -0.075 | -0.476 | 0.322 | 0.720 |
| MAMMALS |  |  |  |  |
| *Unsigned effect sizes* |  |  |  |  |
| Litter size | -0.032 | -0.242 | 0.217 | 0.766 |
| Body mass | 0.000001 | -0.000002 | 0.000005 | 0.415 |
| Captive vs. Wild | -0.232 | -0.945 | 0.570 | 0.539 |
| Experimental vs. Non-experimental | 0.006 | -0.816 | 0.886 | 0.994 |
| Enzymatic antioxidant vs. Non-enzymatic antioxidant | 0.134 | -0.500 | 0.717 | 0.577 |
| Enzymatic antioxidant vs. Oxidation marker | 0.137 | -0.457 | 0.811 | 0.639 |
| Oxidation marker vs. Non-enzymatic antioxidant | 0.013 | -0.710 | 0.565 | 0.886 |
| Adult vs. Mix | 0.930 | -0.575 | 2.673 | 0.251 |
| Adult vs. Young | -0.647 | -1.327 | -0.014 | 0.050 |
| Young vs. Mix | 1.658 | 0.028 | 3.553 | 0.055 |
| *Signed effect sizes* |  |  |  |  |
| Litter size | -0.035 | -0.255 | 0.176 | 0.768 |
| Body mass | 0.000003 | -0.0000003 | 0.000006 | 0.083 |
| Captive vs. Wild | -0.346 | -1.562 | 0.767 | 0.561 |
| Experimental vs. Non-experimental | 0.771 | -0.628 | 2.054 | 0.226 |
| Enzymatic antioxidant vs. Non-enzymatic antioxidant | -0.465 | -1.247 | 0.407 | 0.261 |
| Enzymatic antioxidant vs. Oxidation marker | 0.430 | -0.303 | 1.199 | 0.246 |
| Oxidation marker vs. Non-enzymatic antioxidant | -0.897 | -1.714 | -0.204 | 0.025 |
| Adult vs. Mix | 0.607 | -0.951 | 2.030 | 0.414 |
| Adult vs. Young | 0.346 | -0.780 | 1.432 | 0.528 |
| Young vs. Mix | 0.289 | -1.540 | 1.988 | 0.761 |

Table S10. Models testing the effects of proxies of pace of life (n_birds_ = 253; n_mammals_ = 166): clutch size for birds and litter size for mammals; body mass for both birds and mammals.
